# Dynamical Modulation of Dual‐Band Thermal Emission in a Graphene‐2D‐hBN‐LiV_2_O_5_ Heterostructure

**DOI:** 10.1002/nap2.70180

**Published:** 2026-06-28

**Authors:** Yuhui Cai, Minru Zhu, Peiyuan Huang, Qinmiao Chen, Xuening Wang, Bingfeng Fan, Yongyao Li, Jiayao Huang, Dongxu Zhao

**Affiliations:** ^1^ School of Physics and Optoelectronic Engineering Foshan University Foshan Guangdong China; ^2^ Department of Physics The Hong Kong University of Science and Technology Hong Kong China

**Keywords:** dual‐band thermal emission, plasmon–phonon coupling, Rabi splitting modulation

## Abstract

Dynamic control of thermal emission across multiple infrared bands is crucial for thermal camouflage, molecular sensing, and reconfigurable infrared energy management. However, most emitters rely on broadband, incoherent radiation, limiting spectral precision, and practical tunability. Here, we demonstrate a dynamically tunable dual‐band thermal emission platform based on a van der Waals heterostructure integrating graphene, two‐dimensional hexagonal boron nitride (2D‐hBN) and LiV_2_O_5_. Plasmon–phonon coupling produces five hybrid polariton branches (LP_1_, UP_1_, LP_2_, MP_2_ and UP_2_), enabling narrowband emission in two mid‐infrared windows within a compact stack. To quantify the coupling physics and guide reconfiguration, we develop coupled harmonic oscillator models using two‐mode and three‐mode interaction frameworks. These models enable extraction of Rabi splitting energies, mode composition and strong‐coupling regimes, and they show excellent agreement with numerical simulations. We obtain Rabi splittings of 8.42 meV in the 2D‐hBN channel and 4.32 meV in the LiV_2_O_5_ channel. Increasing the hBN thickness strengthens short‐wavelength confinement and raises the maximum splitting to 27.72 meV. The emitter achieves Q up to 138.97 and $FOM_{\lambda }$ up to 49.33, providing a practical and compact platform for tunable mid‐infrared sources for adaptive thermal control, molecular detection, and multispectral imaging.

## Graphical abstract

A van der Waals heterostructure combining graphene, 2D‐hBN, and LiV_2_O_5_ enables dynamically tunable dual‐band mid‐infrared thermal emission with narrow spectral features. Plasmon–phonon strong coupling yields five hybrid polariton branches, whereas coupled‐oscillator models quantify mode mixing and Rabi splittings (up to 27.72 meV via hBN‐thickness control), pointing to compact sources for adaptive thermal control and sensing.

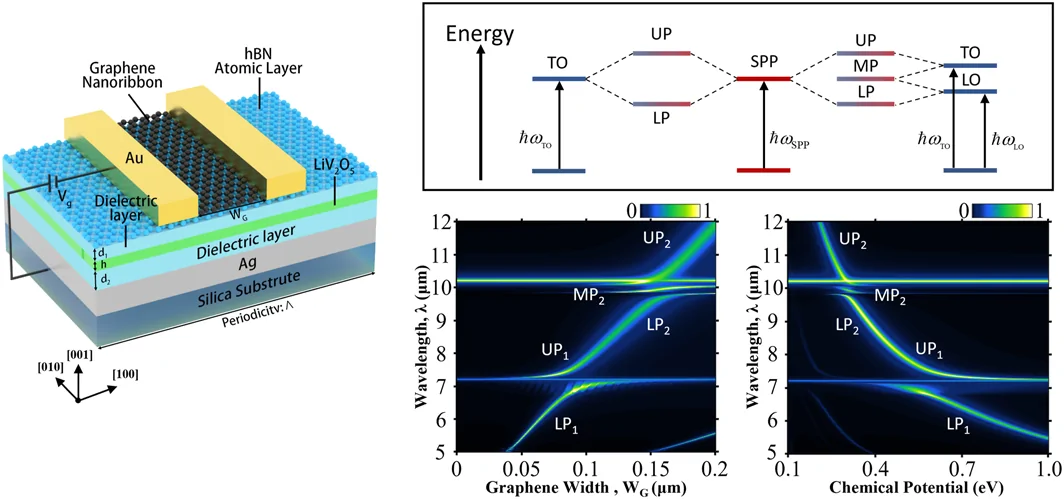

## 1 Introduction

Thermal emission, driven by thermally induced electromagnetic fluctuations, governs radiative heat transfer and photon‐based energy conversion. Its broadband, isotropic and incoherent nature, however, hampers applications that require tailored directionality, spectral response or polarization. Dynamic and real‐time modulation of thermal radiation has therefore become central to adaptive thermal management [1], multispectral infrared imaging [2], and nonreciprocal thermal photonics [3]. Graphene is particularly appealing for such active control because it combines a strong electro‐optic response with low thermal mass and excellent integration compatibility. Electrical tuning of its Fermi level, together with nanostructure engineering [4], enables fast and reversible mid‐infrared emission control with spectral selectivity [5, 6], supporting dynamically reconfigurable thermal photonic systems. Although much of the early work concentrated on single‐band modulation, emerging applications increasingly demand dual‐band operation to address separated infrared windows within one device [7, 8]. Existing dual‐band emitters remain largely confined to static designs with emission bands fixed after fabrication [7, 9, 10], or phase‐change‐based implementations that switch between discrete emissive states [8, 11, 12]. As a result, dual‐band thermal emission that is both continuously tunable and independently controllable remains elusive.

A central requirement for independently controllable dual‐band emission is a pair of polaritonic channels that are spectrally separated. Two‐dimensional hexagonal boron nitride (2D‐hBN) satisfies this requirement in the short‐wavelength mid‐infrared by supporting low‐loss transverse optical (TO) phonons and narrowband thermal features near 7 μm, whereas anisotropic LiV_2_O_5_ complements it with broadband TO and longitudinal optical (LO) phonon responses around 10–12 μm [13, 14]. This combination provides two distinct phonon‐defined windows that can be engineered as separate emission channels within one platform. Both materials are fabrication compatible with van der Waals integration and multilayer infrared optical stacks. The hBN flakes can be obtained by mechanical exfoliation and assembled through mature transfer and encapsulation routes [15, 16]. In addition, the hBN is widely used as an atomically smooth buffer that helps retain graphene mobility and narrow‐linewidth plasmonic responses in complex stacks. Thickness controlled LiV_2_O_5_ films can be produced by exfoliating flux grown crystals [14]. Their bulk phonon‐driven infrared response enables a continuous, unpatterned interlayer that preserves robust Reststrahlen band characteristics on dielectric or metal substrates. These phononic platforms further enable coherence engineering through polaritonic mode coupling. Spatial coherence can be improved via Surface Phonon Polariton (SPhP) [17], Surface Plasmon Polariton (SPP) [18], and hybrid modes, and temporal coherence is set by linewidth and benefits from spectral narrowing [19]. Coherence tailoring has been demonstrated using grating resonators, subwavelength grooves and photonic crystals structures [20]. Combining tunable plasmonic elements with phonon‐active media has emerged as a route to spectrally selective, mode‐defined thermal emission [21, 22].

In this work, we realize a continuously and reversibly reconfigurable dual‐band thermal emitter by integrating graphene nanoribbons with 2D‐hBN and LiV_2_O_5_ in a van der Waals heterostructure. The design exploits the spectrally separated phonon resonances of hBN and LiV_2_O_5_ to address two atmospheric transparency windows near 7 and 10 μm within a single compact platform. Graphene provides active control through electrostatic gating, which tunes the plasmon dispersion, whereas the nanoribbon geometry offers an independent handle that regulates near‐field confinement and symmetry matching. This architecture enables channel‐resolved control over both emissivity strength and resonance position, directly targeting the need for independent and continuous dual‐band modulation highlighted above. We model the thermally excited spectral response and field‐phonon interactions using rigorous coupled‐wave analysis (RCWA), and use coupled‐oscillator descriptions to track modal evolution and identify systematic modulation trends governed by graphene's Fermi level and ribbon geometry. To compare all resolved branches on equal footing, we quantify performance with a unified set of metrics, including the quality factor (Q), modulation contrast ($C_{\varepsilon }$ and $R_{\varepsilon }$), and figures of merit ($FOM_{\lambda }$). These results establish a dual‐band mid‐infrared emissivity platform that is tunable, reversible, and compact, providing a route towards adaptive dual‐band thermal photonics.

## 2 Models and Principle

The dual‐band emission platform in Figure 1a is engineered for dynamic thermal radiation control in the mid‐infrared. The van der Waals heterostructure consists of graphene nanoribbons, atomically thin 2D‐hBN, and anisotropic LiV_2_O_5_ layers, integrated with BaF_2_ spacers for optical isolation and a metallic Ag back‐reflector to enhance emissive out‐coupling. We determine the BaF_2_ spacer thicknesses $d_{1}$, $d_{2}$, and the grating periodicity Λ using physics guided RCWA parametric optimization. The design places hybrid plasmon–phonon resonances in the atmospheric windows near 7 and 10 μm and strengthens the Rabi splitting features. Spacer thickness $d_{1}$ sets the evanescent field overlap between graphene plasmons and LiV_2_O_5_ phonons, and $d_{2}$ reduces Ag back reflector dissipation while maintaining field confinement in the active layers. The grating periodicity Λ supplies the in‐plane momentum that couples normally incident radiation into graphene plasmons and therefore defines the hybridization condition. Throughout this work, the geometric parameters are fixed at $d_{1}=50\;\text{nm}$, $d_{2}=1200\;\text{nm}$, and Λ=300nm. Two phonon‐mediated emission channels enable spectrally selective modulation. These two phonon‐mediated emission channels remain spectrally independent across the full 6–12 μm window, as verified in Supporting Information S1: Section S1, with the hBN channel yielding narrowband emission near 7 μm and the LiV_2_O_5_ channel providing richer spectral features and tunability around 10 μm. Spectral emissivity $\varepsilon_{\lambda }$ is derived from reflectivity $R_{\varepsilon }$ using Kirchhoff's law, as $\varepsilon_{\lambda }=\alpha_{\lambda }=1-R_{\lambda }$, under thermal equilibrium conditions [23, 24].

**FIGURE 1:**
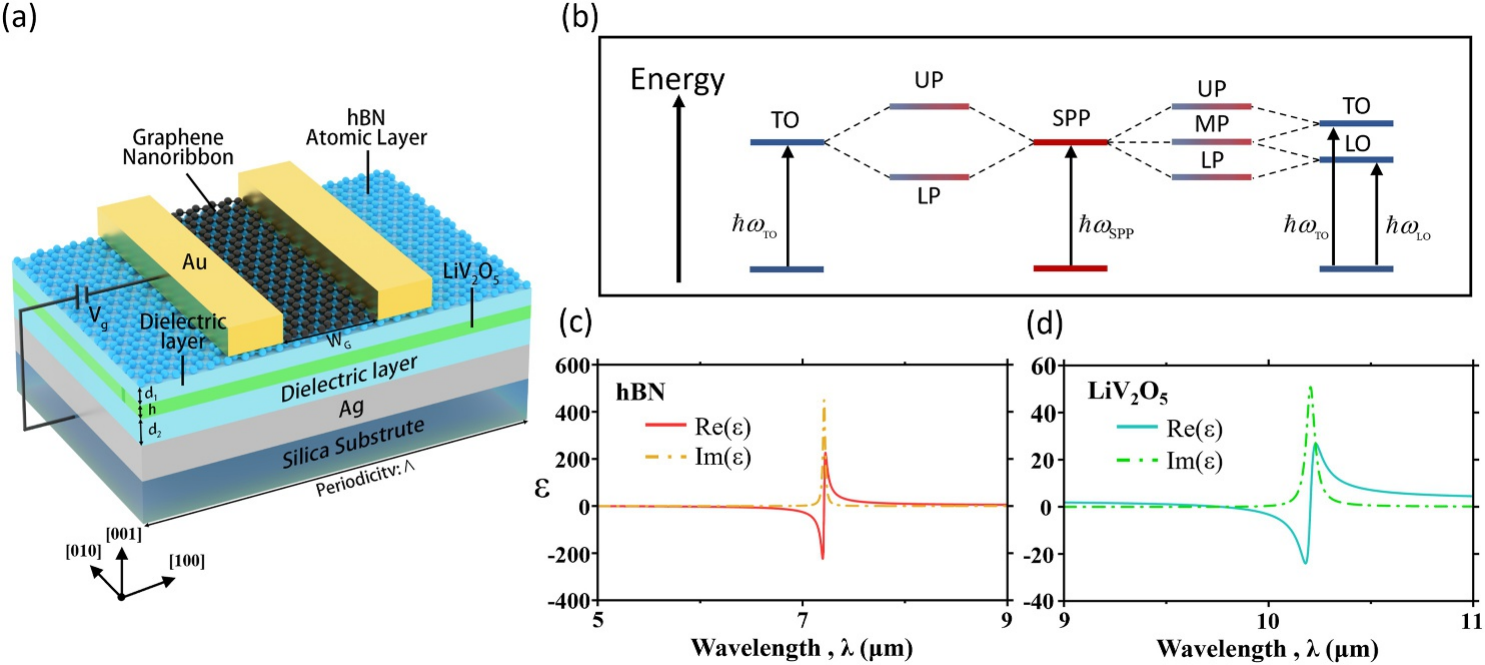
Dynamically tunable dual‐band thermal emission platform based on a graphene–2D‐hBN–LiV_2_O_5_ heterostructure. (a) Schematic of the device integrating graphene nanoribbons, monolayer 2D‐hBN, and LiV_2_O_5_ thin films, separated by low‐loss dielectric spacers and supported by a reflective Ag substrate. The structure is optimized for mid‐infrared thermal emission control near 7 and 10 μm through modulation of Λ, $W_{G}$, h, $d_{1}$ and $d_{2}$. (b) Energy‐level diagrams illustrating hybridization mechanisms: left, interaction between graphene SPP and 2D‐hBN TO phonon gives rise to upper and lower emissive branches; right, additional coupling to TO and LO phonons of LiV_2_O_5_ introduces a middle emissive channel. (c, d) Real and imaginary parts of the permittivity $\varepsilon_{\lambda }$ for 2D‐hBN and LiV_2_O_5_.

As shown in Figure 1b, the dual‐band thermal emission response arises from two phonon–plasmon hybridization channels. The first originates from the interaction between graphene SPP and the TO phonon of 2D‐hBN at a wavelength of 7 μm, yielding two emissive branches corresponding to the upper and lower hybridized modes [25]. The second involves the simultaneous coupling between graphene SPP and both TO and LO phonons of LiV_2_O_5_ at a wavelength around 10 μm. This three‐channel hybridization leads to the formation of three distinct emissive polariton branches, namely lower (LP), middle (MP), and upper (UP). Conventional dual‐band emitters combine distinct resonators or switch between discrete phase change states. In our graphene–2D‐hBN–LiV_2_O_5_ platform, both bands are independently tuned in a continuous and reversible manner through electrostatic gating, providing real time spectral control without moving components or phase transitions [8, 9, 26].

Figure 1c,d show wavelength‐dependent complex permittivity of 2D‐hBN and LiV_2_O_5_ along the out‐of‐plane direction, where real and imaginary parts are indicated by solid and dashed lines. In monolayer 2D‐hBN, the infrared response is set by a dispersive complex permittivity and is dominated by a TO phonon at 7.2 μm. This resonance defines the Type II Reststrahlen band spanning 6.2–7.3 μm and supports an SPhP‐like channel at the graphene–hBN interface [27, 28]. The LO phonon at 6.2 μm has negligible out of plane dipole activity and therefore contributes little to infrared emission. By contrast, LiV_2_O_5_ exhibits both TO and LO phonon modes along the a‐ and c‐axes, whereas the b‐axis response is comparatively weak. For the c‐axis response used in this work, these phonon modes define a Reststrahlen band from approximately 9.8–10.2 μm, where the real permittivity becomes negative [14]. This region enables strong confinement of electromagnetic energy through phonon‐supported modes with low losses. Both the SPhP in hBN and the phonon polaritons in LiV_2_O_5_ have been directly imaged in recent near‐field experiments, corroborating the physical reality of the polaritonic channels used here [14, 28]. The dielectric function and surface conductivity of monolayer 2D‐hBN are described by [18, 26]:

(1) $$\varepsilon_{\text{hBN},\text{mono}}=1+i\frac{\sigma_{\text{hBN},\text{mono}}}{\varepsilon_{0}\omega N_{\text{hBN},\text{mono}}\alpha_{\text{hBN},\text{mono}}}$$

(2) $$\sigma_{\text{hBN},\text{mono}}=-i\frac{4\varepsilon_{0}\omega \omega_{\text{TO},\text{mono}}\upsilon_{g}}{\omega_{\text{TO},\text{mono}}^{2}-\omega^{2}-i\omega \gamma_{\text{mono}}}$$

where ω denotes the angular frequency and $\varepsilon_{0}$ is the vacuum permittivity. The number of 2D‐hBN layers and their total thickness are denoted by $N_{\text{hBN},\text{mono}}=1$ and $\alpha_{\text{hBN},\text{mono}}=3.4Å$, respectively. The anisotropic permittivity of 2D‐hBN $\sigma_{\text{hBN},\text{mono}}$ is obtained via first‐principles calculations, incorporating material‐specific parameters such as the TO phonon frequency $\omega_{\text{TO},\text{mono}}=1387\;{\text{cm}}^{-1}$, damping coefficient $\gamma_{\text{mono}}=5\;{\text{cm}}^{-1}$, and phonon group velocity $v_{\pi }=1.2\times 10^{-4}\;c_{0}$. Here, $c_{0}$ is the speed of light in vacuum. The complex permittivity of LiV_2_O_5_ is described by the Drude–Lorentz formalism as follows [14]:

(3) $$\varepsilon_{{\text{LiV}}_{2}O_{5},i}=\varepsilon_{\infty }^{i}\prod_{j=1}^{N_{i}}\left(\frac{{\left(\omega_{\;\text{LO},j}^{i}\right)}^{2}-\omega^{2}-i\gamma_{\text{LO},j}^{i}}{{\left(\omega_{\;\text{TO},j}^{i}\right)}^{2}-\omega^{2}-i\gamma_{\text{TO},j}^{i}}\right)$$

where the high‐frequency permittivity are $\varepsilon_{\infty }^{a}=6.28$, $\varepsilon_{\infty }^{b}=4.48$, and $\varepsilon_{\infty }^{c}=2.71$. Table 1 presents the TO and LO phonon frequencies along with their corresponding damping rates. The first column specifies the crystallographic axis (a, b, or c), and the second indices the *j*th oscillator along the *i*th direction. Owing to the material's strong anisotropy, we use the c‐axis dielectric function as the representative case in the main text. It yields a well‐defined long‐wavelength Reststrahlen channel that is separated from the hBN band by more than 2 μm, which suppresses interlayer phonon–phonon coupling and enables independent dual‐band operation. Emission for other crystallographic orientations is reported in Supporting Information S1: Section S2. In this c‐axis Reststrahlen band of LiV_2_O_5_, 2D‐hBN functions mainly as a low‐loss dielectric overlayer. The negligible hBN–LiV_2_O_5_ interlayer coupling is validated in Supporting Information S1: Section S3. Graphene's dielectric response can be dynamically tuned via electrostatic gating and carrier doping, thereby enabling selective control of dual‐band mid‐infrared emissivity in the graphene–2D‐hBN–LiV_2_O_5_, heterostructure. Its dielectric function is modeled using an effective medium approximation capturing its linear band dispersion and electronic structure [18, 29, 30]:

(4) $$\varepsilon_{G\;}=1+i\frac{\sigma_{G}}{\varepsilon_{0}\;\omega N_{G}a_{G}}$$

where ω is the angular frequency and $\varepsilon_{0}$ is the vacuum permittivity. Furthermore, $N_{G}$ and $a_{G}$ are denoted as the numbers and thicknesses of graphene, where $a_{G}$ is 3.3 Å. The $\varepsilon_{G}$ and $\sigma_{G}$ correspond to the permittivity and sheet conductivity. The sheet conductivity of graphene is described using the Kubo formula, incorporating both intraband ($\sigma_{\text{intra}}$) and interband ($\sigma_{\text{inter}}$) transition contributions [29]:

(5) $$\begin{array}{c} \sigma_{G}=\sigma_{\text{intra}}+\sigma_{\text{inter}}\\ =i\frac{e^{2}k_{B}T}{\pi \hbar^{2}\left(\omega +i\tau^{-1}\right)}\left[\frac{\mu_{c}}{k_{B}T}+2\;\ln\left(\exp\left(\frac{\mu_{c}}{k_{B}T}\right)+1\right)\right]\\ +i\frac{e^{2}}{4\pi \;\hbar }\;\ln\;\left[\frac{2|\mu_{c}|-\hbar \left(\omega +i\tau^{-1}\right)}{2\left|\mu_{c}\right|+\hbar \left(\omega +i\tau^{-1}\right)}\right] \end{array}$$

**TABLE 1:** Parameters of the IR dielectric function of LiV_2_O_5_ [14].

Crystal axis (i)	Oscillator index (j)	$\omega_{\text{TO}}$ (cm^−1^)	$\gamma_{\text{TO}}$ (cm^−1^)	$\omega_{\text{LO}}$ (cm^−1^)	$\gamma_{\text{LO}}$ (cm^−1^)
a	1	483.1	27.9	625.2	1.8
a	2	731	149	738.9	168.6
a	3	948.4	5.5	963.9	9.1
a	4	1004.9	6	1011.5	8.5
b	1	513.3	21.6	573.3	13.9
b	2	581	26.3	795.4	18.6
c	1	980	4.8	1024	4.8

Here, e represents the electron charge, $k_{B}$ is the Boltzmann constant, and ℏ is the reduced Planck's constant. The temperature is set to 300 K, and the carrier relaxation time is defined as $\tau =\mu \mu_{c}/e{v_{\text{F}}}^{2}$, where $v_{\text{F}}=10^{6}m/s$ is the Fermi velocity, and $\mu =10^{4}\text{cm}/\left(V\cdot \;s\right)$ represents the carrier mobility [30]. The Fermi level $\mu_{\text{c}}$ is controlled by the carrier density $n_{s}$ [31]. The dynamic interplay between tunable graphene plasmons and the phonon modes in 2D‐hBN and LiV_2_O_5_ governs the spectral position and coupling strength of the hybrid mode.

## 3 Result and Discussion

Figure 2 establishes tunable dual‐band polaritonic channels that enable modulation of thermal emission in the graphene–2D‐hBN–LiV_2_O_5_ platform. We excite graphene nanoribbon SPP using TM‐polarized plane of the LiV_2_O_5_ layer, which can support slab‐guided phonon–polariton components in analogy with other finite polar slabs [14, 28, 32]. Its stronger visibility under $W_{G}$ tuning is consistent with the sensitivity of graphene–polar‐material hybrid modes to plasmon momentum, lateral confinement and field‐overlap conditions [33, 34]. A comparison with representative dual‐band thermal emitters is provided in Supporting Information S1: Section S4.

**FIGURE 2:**
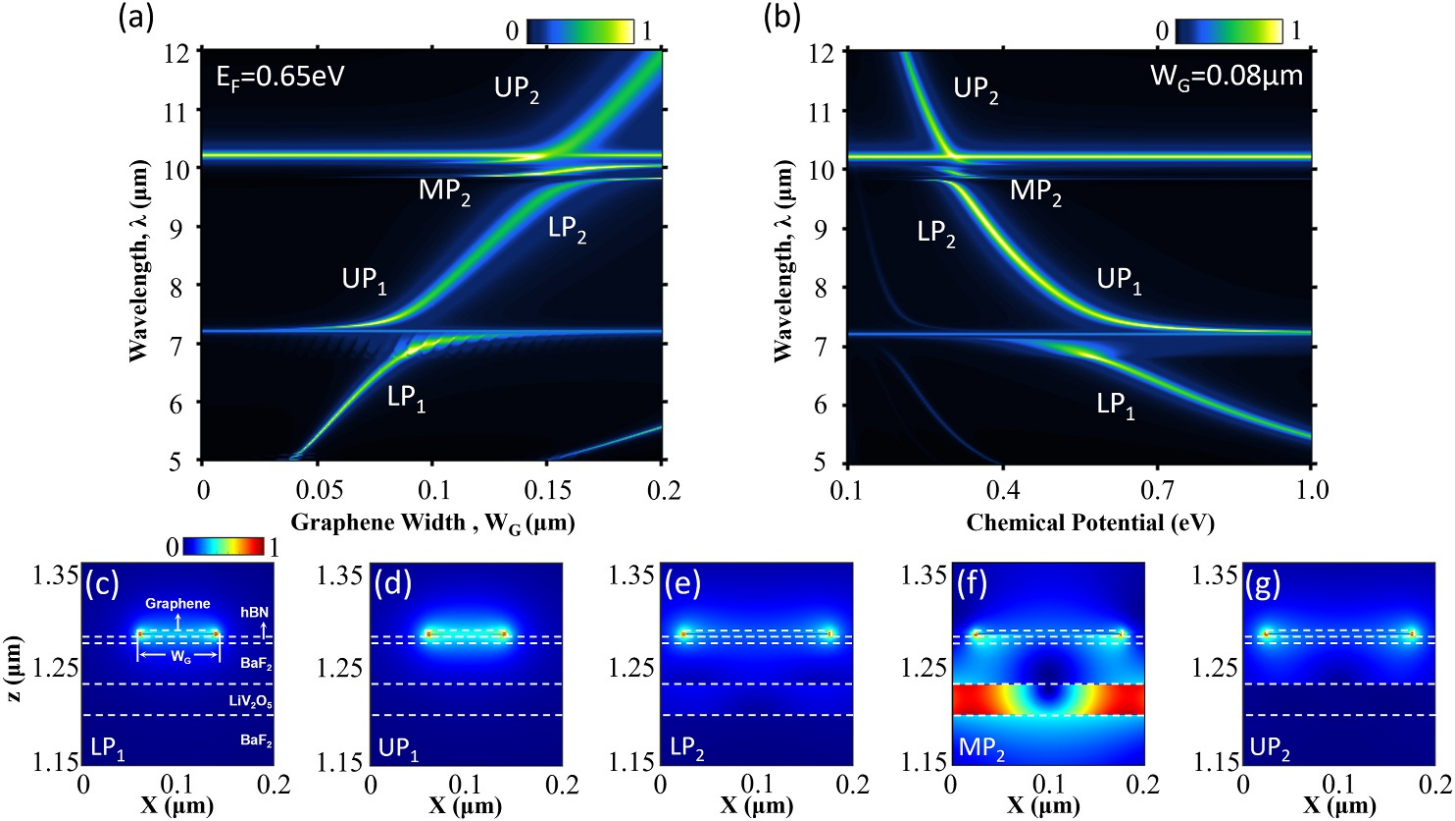
Tunable dispersion and near‐field confinement in a dual‐band thermal emission platform. (a, b) Simulated dispersion diagrams under varying graphene nanoribbon width $W_{G}$ (at fixed Fermi energy $E_{F}=0.65\;\text{eV}$) and chemical potential $E_{F}$ (at fixed width $W_{G}=0.08\;\mu m$), respectively. The observed spectral shifts near 7 and 10 μm correspond to dynamic modulation of hybridized modes involving graphene SPP and phonons in 2D‐hBN and LiV_2_O_5_. The formation of five distinct branches, including LP_1_, UP_1_, LP_2_, MP_2_, and UP_2_, highlights the tunability of thermal emission. (c–g) Corresponding electric field distributions in the X–Z plane, illustrating the spatial confinement and mode symmetry of the hybrid polaritons ($W_{G}$ is the nanoribbon width).

The hybrid resonances translate into strong emissivity enhancement. The peak emissivity approaches unity at the polaritonic resonances and the constituent‐only baselines, as quantified by blackbody‐referenced benchmarks in Supporting Information S1: Section S5. We further verify the modal character through near‐field profiles in the X‐Z plane, as shown in Figure 2c–g. The dominant confinement appears across the ribbon width and is consistent with short‐edge confinement [35]. The LP_1_ and UP_1_ modes in Figure 2c,d are localized at the graphene hBN interface, whereas the LP_2_, MP_2_, and UP_2_ modes in Figure 2e–g exhibit pronounced confinement inside the phonon‐active region of LiV_2_O_5_. The edge‐enhanced intensity originates from boundary‐induced charge accumulation in width‐quantized plasmon eigenmodes [36]. In our heterostructure, this edge‐weighted localization increases the overlap with the phonon‐active layers and reduces the effective modal area, which reinforces plasmon–phonon hybridization and supports the tunability of the LP, UP, and MP branches in Figure 2a,b. We note that the edge enhancement reflects the intrinsic spatial structure of the hybrid eigenmodes rather than parasitic edge‐radiative channels. This lateral mode selectivity, combined with longitudinal uniformity, supports spectrally selective emission and may improve spatial coherence [24].

Figure 3 establishes that the emission response near 7 μm is governed by plasmon–phonon polaritons formed inside the hBN Type II Reststrahlen band instead of separate graphene or hBN resonances. Figure 3a identifies a single sharp resonance at 7.2 μm in agreement with the infrared‐active TO phonon from monolayer 2D‐hBN. Introducing graphene transforms this isolated feature into a pair of narrow peaks near 7 μm. The emergence of the UP and LP branches indicates coupling‐induced splitting. This interpretation is reinforced by the dispersion behavior. Figure 3b,c show a clear anticrossing within 6–8 μm under both ribbon‐width tuning and electrostatic tuning, providing unambiguous evidence for strong plasmon–phonon hybridization enabled by the Reststrahlen band polaritonic channel. To analyze the energy exchange dynamics and mode hybridization, we apply a coupled two‐harmonic oscillator model [37]:

(6) $$\left[\begin{array}{cc} \hbar \omega_{\text{SPP}}-i\hbar \frac{\gamma_{\text{SPP}}}{2} & g\\ g & \hbar \omega_{\text{TO}}-i\hbar \frac{\gamma_{\text{TO}}}{2} \end{array}\right]\left[\begin{array}{l} \alpha_{\text{SPP}}\\ \alpha_{\text{TO}} \end{array}\right]=\hbar \omega \left[\begin{array}{l} \alpha_{\text{SPP}}\\ \alpha_{\text{TO}} \end{array}\right]$$

where $\gamma_{\text{SPP}}$ and $\gamma_{\text{TO}}$ denote the linewidths of the graphene plasmon and hBN TO phonon modes, respectively. The coupling strength g quantifies the interaction between these two resonances. The eigenvectors $\alpha_{\text{SPP}}$ and $\alpha_{\text{TO}}$ satisfy the normalization condition ${\left|\alpha_{\text{SPP}}\right|}^{2}+{\left|\alpha_{\text{TO}}\right|}^{2}=1$, with their squared magnitudes representing the mixing fractions in the hybrid states. The $\hbar \omega_{\text{SPP}}$ and $\hbar \omega_{\text{TO}}$ correspond to the uncoupled mode energies. The eigenvalues $\hbar \omega_{\pm }$ of the coupled system are given by Section 7.

**FIGURE 3:**
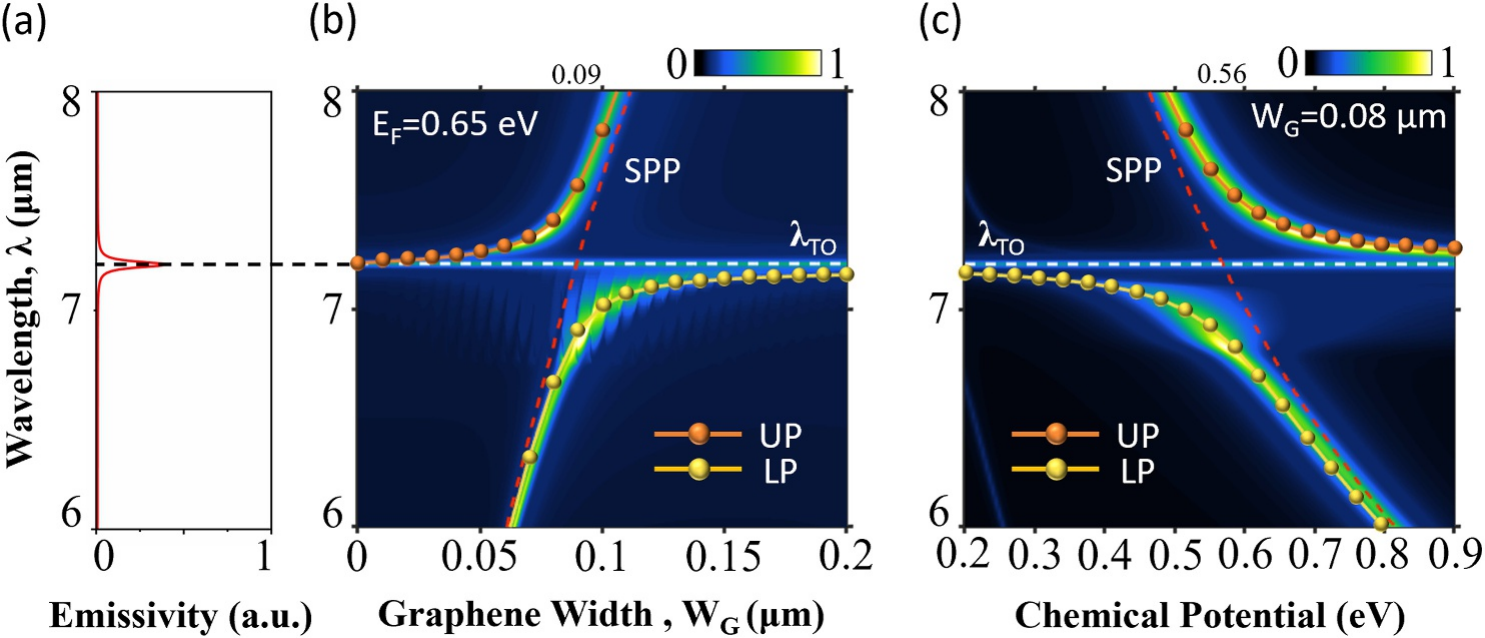
Tunable polariton dispersion and emissivity near the 2D‐hBN TO phonon resonance. (a) Emissivity spectrum of monolayer 2D‐hBN, highlighting the TO phonon resonance. (b) Dispersion map within the 6–8 μm range showing the evolution of polariton modes as a function of graphene nanoribbon width $W_{G}$ at a fixed chemical potential $E_{F}=0.65\;\text{eV}$. (c) Dispersion map over the same spectral range, illustrating mode tuning via graphene chemical potential $E_{F}$ at a fixed width $W_{G}=0.08\;\mu m$. Solid orange and yellow lines represent the UP and LP polariton branches, respectively, fitted using the two‐harmonic oscillator model.

Figure 4 provides a detailed analysis of the modulation behavior in the graphene–2D‐hBN system, focusing on spectral splitting, resonance evolution, and intermodal energy redistribution under structural and electronic tuning. For blackbody‐referenced emission benchmarks and layer‐resolved emissivity comparisons (isolated graphene, phonon‐layer, and the coupled heterostructure) for both channels at *T* = 300 K, refer to Fig. S4. At the anticrossing point ($\hbar \omega_{\text{SPP}}=\hbar \omega_{\mathrm{TO}}$) identified in Figure 4a, the LP and UP branches exhibit a clear spectral separation with a splitting energy of $\Omega_{0}=8.42\;\text{meV}$. Using the two‐harmonic oscillator model, the linewidths of the bare SPP mode and TO phonon modes are extracted as $\gamma_{\text{SPP}}=5.64\;\text{meV}$ and $\gamma_{\text{TO}}=0.72\;\text{meV}$, yielding a more interaction strength of g=4.50meV through the relation:

(7) $$\Omega_{0}=\hbar \omega_{+}-\hbar \omega_{-}=\sqrt{4g^{2}-\frac{{\left(\gamma_{\text{SPP}}-\gamma_{\text{TO}}\right)}^{2}}{4}}$$

**FIGURE 4:**
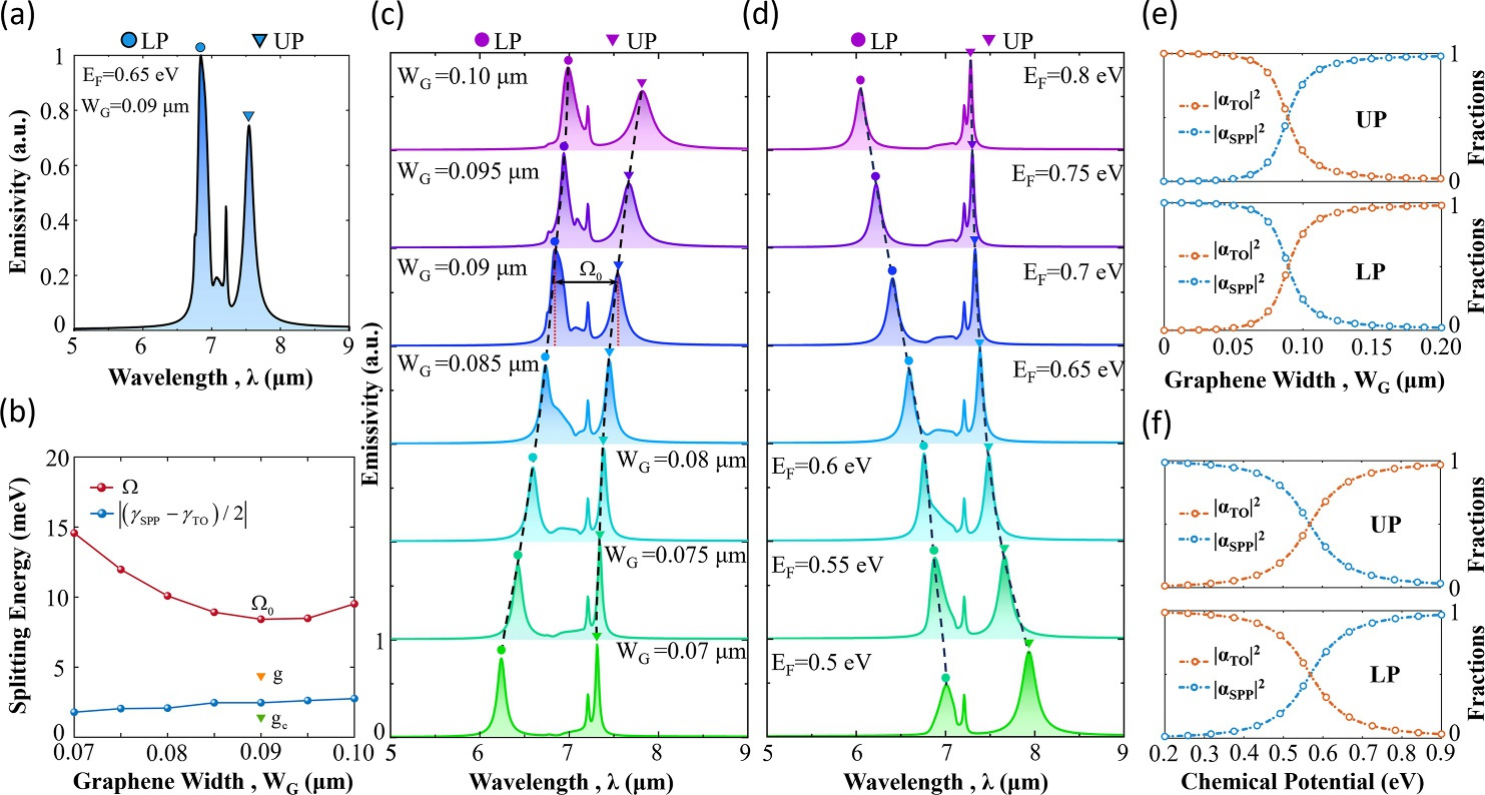
Dynamical modulation of thermal emission near the 2D‐hBN phonon resonance. (a) Emissivity spectrum at $W_{G}=0.08\;\mu m$ and $E_{F}=0.65\;\text{eV}$, showing distinct LP and UP emission features. (b) Extracted spectral splitting energy Ω, plasmon–phonon detuning threshold $\left|\left(\gamma_{\text{SPP}}-\gamma_{\text{TO}}\right)/2\right|$, the effective modulation strength g and the critical coupling threshold $g_{c}$ as a function of graphene nanoribbon width $W_{G}$. (c, d) Emission spectra under variable graphene width $W_{G}$ (at fixed $E_{F}=0.65\;\text{eV}$) and chemical potential $E_{F}$ (at fixed $W_{G}=0.08\;\mu m$), respectively, demonstrating continuous spectral reshaping. (e, f) Mode composition evolution of LP and UP branches with respect to $W_{G}$ and $E_{F}$, respectively, illustrating tunable energy distribution between graphene plasmons and hBN phonons.

To determine whether the system reaches a modulation regime dominated by hybrid mode rather than perturbative spectral overlap, we evaluate two standard criteria [38, 39]:

(8) $$g>\frac{\left|\gamma_{\text{SPP}}-\gamma_{\text{TO}}\right|}{2}$$

(9) $$g>g_{c}=\frac{\sqrt{{\gamma_{\text{SPP}}}^{2}+{\gamma_{\text{TO}}}^{2}}}{4}$$

Here, $g_{c}=4.50\;\text{meV}$ denotes the critical coupling threshold. For the case in Figure 4a, both conditions are fulfilled ($\left|\left(\gamma_{\text{SPP}}-\gamma_{\text{TO}}\right)/2\right|=2.46\;\text{meV}$ and $g_{c}=1.42\;\text{meV}$), suggesting that field confinement and mode overlap play dominant roles in shaping the emission spectrum. To further assess the tunability of this interaction, we investigate the evolution of Ω with graphene nanoribbon width $W_{G}$ in Figure 4b. A clear nonmonotonic trend emerges as the splitting energy decreases from 14.57 meV to a minimum of 8.42 meV around $W_{G}=0.09\;\mu m$ and then increases again. Figure 4c,d demonstrate dynamic and reversible control of the polaritonic branches near the 2D‐hBN phonon resonance. Both nanoribbon width modulation and electrostatic gating reconfigure the LP and UP branches, which enables branch‐selective reshaping of the emissivity spectrum. To quantify this behavior, we evaluate three standardized metrics for all configurations, including the quality factor Q, the modulation contrasts $C_{\varepsilon }$ and $R_{\varepsilon }$, and the figures of merit $FOM_{\lambda }$. Definitions are provided in Supporting Information S1: Section S8. In the graphene‐2D‐hBN channel, the quality factors reach 49.50 for the LP branch and 95.78 for the UP branch. The same platform offers substantial agility, with $FOM_{\lambda }$ ranging from 1.95 to 13.29 under geometric and electrostatic tuning. The emissivity redistribution is branch‐selective and depends on the tuning route. Under geometric modulation, the most evident change is the suppression of the UP branch, with $C_{\varepsilon }=-0.25$ and $R_{\varepsilon }=0.75$, whereas the LP branch remains nearly unchanged. Under electrostatic gating, the LP branch shows the stronger enhancement, with $C_{\varepsilon }=0.18$ and $R_{\varepsilon }=1.18$. These representative values indicate that geometric tuning mainly redistributes emission between the two hybrid branches, whereas electrostatic gating provides a more direct enhancement of the lower branch. Complete statistics are listed in Supporting Information S1: Tables S2 and S3. We interpret these trends by analyzing the hybrid eigenmodes obtained from Equation (6). The eigenvector‐derived mixing fractions are calculated using Supporting Information S1: Formula S4 and S5. Figure 4e,f reveal a continuous exchange of modal character under both tuning routes. Increasing the graphene width drives the LP branch from SPP‐like to phonon‐like behavior, and drives the UP branch in the opposite direction. The crossover occurs close to the point of minimal branch separation, indicating maximal hybridization. Varying the Fermi level induces a complementary evolution. The LP branch becomes more SPP‐like whereas the UP branch becomes more phonon‐like. The ability to reversibly steer these mixing fractions provides a practical mechanism to reconfigure infrared emission by redistributing energy between plasmonic and phononic components, which directly impacts the emission spectrum and its coherence.

Figure 5 investigates the impact of multilayer 2D‐hBN on the thermal emission characteristics and spectral modulation of the graphene–2D‐hBN–LiV_2_O_5_ heterostructure. Before discussing the thickness‐dependent spectra, we first clarify the hBN dielectric modeling scheme. The layer‐dependent dielectric constants of hBN indicate that the out‐of‐plane dielectric response evolves continuously from *N* = 2 to *N* = 4 and becomes nearly saturated above *N* = 5 [40, 41]. Therefore, *N* = 5 is treated as the onset of the bulk regime, whereas *N* = 2 to 4 is regarded as an intermediate quasi‐bulk regime. Accordingly, the monolayer sheet model is used for *N* = 1, the layer‐dependent 3D anisotropic thin‐film model is used for *N* = 2 to 4, and the bulk‐like 3D anisotropic model is used for *N* ≥ 5 [42]. The difference between the monolayer model and the 3D anisotropic model is examined in Supporting Information S1: Section S10.

**FIGURE 5:**
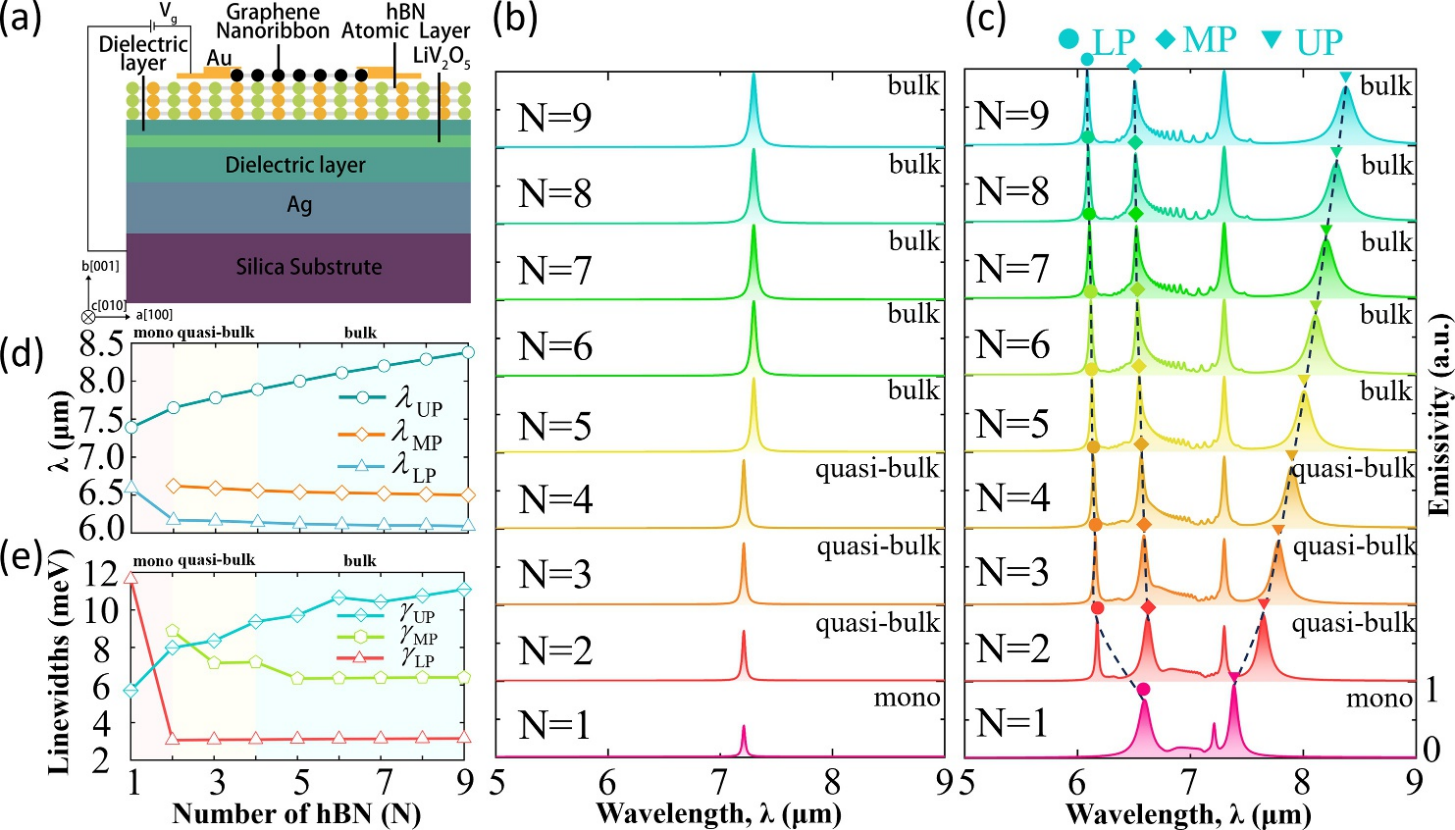
Multilayer 2D‐hBN effects on dual‐band thermal emission and spectral modulation. (a) Gated graphene nanoribbon/multilayer hBN/LiV_2_O_5_ heterostructure with hBN layer number *N*. (b) RCWA emissivity without graphene for *N* = 1 to 9 (mono model for *N* = 1, quasi‐bulk for *N* = 2 to *N* = 4, and bulk for *N* ≥ 5). (c) RCWA emissivity with graphene, showing LP/UP and the emergence of an MP branch as *N* increases. (d, e) Extracted resonance wavelengths ($\lambda_{\text{LP}}$, $\lambda_{\text{MP}}$, $\lambda_{\text{UP}}$) and linewidths ($\gamma_{\text{LP}}$, $\gamma_{\text{MP}}$, $\gamma_{\text{UP}}$) versus the number of hBN (*N*).

To highlight the thickness effect, Figure 5b,c compare the thermal emissivity spectra for hBN layer number *N* = 1 to 9 in the absence and presence of graphene. In Figure 5b, without graphene, the spectrum is dominated by a resonance near the hBN TO phonon around 7.2 μm. As the number of hBN layers increases, this resonance becomes sharper and stronger, whereas its spectral position remains nearly unchanged. This trend is consistent with the gradual strengthening of the hBN dielectric response from the monolayer to the multi‐layer regime. After introducing graphene, Figure 5c shows that the hBN phonon resonance is transformed into several SPP–phonon hybrid resonances. For *N* = 1, the spectrum is mainly characterized by a hybrid peak near 7.5 μm, together with narrow phonon‐related features around the hBN phonon region. When the hBN thickness increases to *N* = 2 to 4, the LO‐related channel begins to participate in the hybridization process, leading to a rapid reorganization of the hybrid spectrum. The LP branch shifts towards approximately 6.0 μm and becomes substantially narrower, whereas the MP branch remains near 6.4–6.6 μm with only a weak spectral displacement. The UP branch shifts continuously from around 7.5 μm towards 8.3–8.5 μm as *N* increases. Meanwhile, the narrow hBN phonon‐like feature near 7.2 μm persists and becomes more pronounced as the hBN layer number increases. For *N* ≥ 5, the dielectric constants of hBN are nearly saturated, and the spectra gradually approach the bulk regime, where the main hybrid branches evolve more smoothly with thickness. The maximum Rabi splitting energy reaches 27.72 meV in *N* = 9.

Figure 5d,e summarize the extracted resonance positions and linewidths of the hybridized modes as a function of hBN layer number. As *N* increases, the separation between the hybrid branches becomes larger, confirming that thicker hBN layers strengthen the SPP–phonon interaction. The linewidths of the LP, MP, and UP branches show different trends. The LP linewidth decreases rapidly after the monolayer case and then remains relatively narrow. The MP branch keeps a small linewidth over the full thickness range. In contrast, the UP linewidth increases more clearly with *N*, indicating stronger plasmonic participation and stronger radiative broadening in thicker hBN structures. As discussed in Supporting Information S1: Section S10, the abrupt change of $\gamma_{\text{LP}}$ in the few‐layer region mainly originates from the dielectric‐model transition and the corresponding branch reassignment. Having clarified the hBN‐thickness effect, we next examine the complementary graphene–LiV_2_O_5_ channel in Figure 6.

**FIGURE 6:**
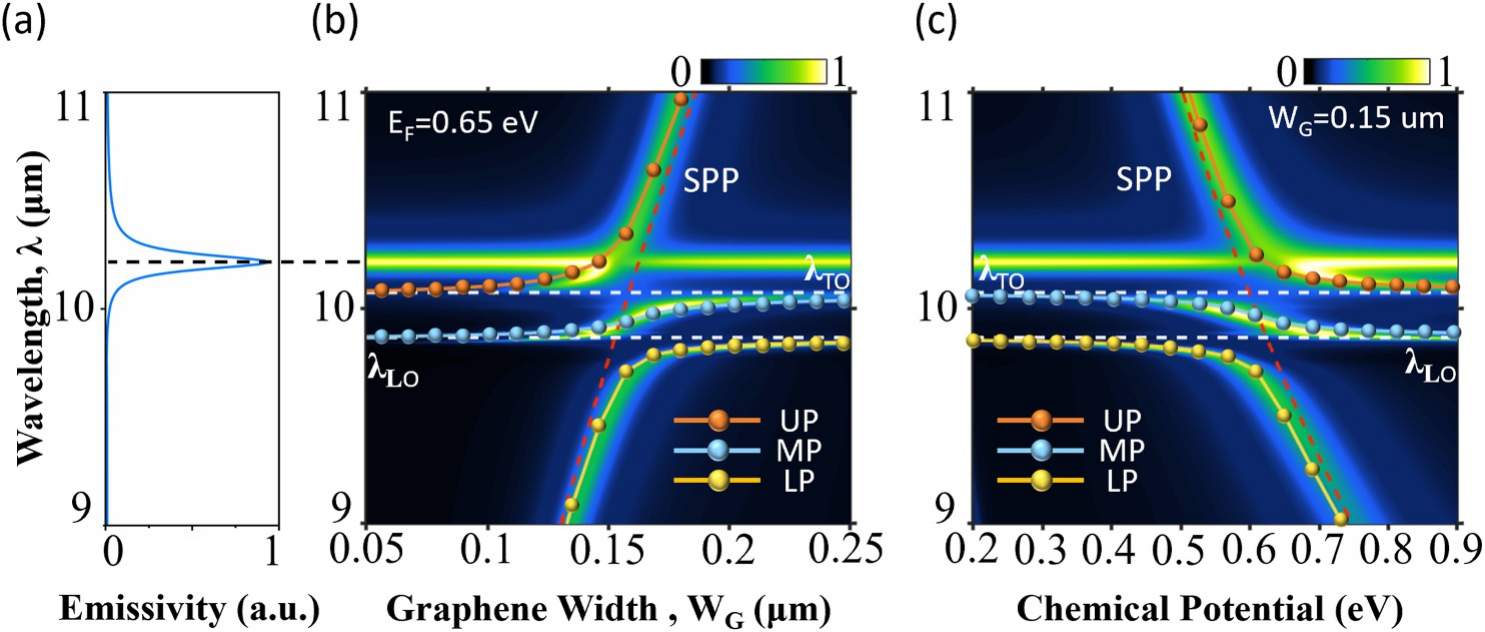
Dual‐band thermal emission and dynamic modulation in the graphene–LiV_2_O_5_ system. (a) Emissivity spectrum of bare LiV_2_O_5_, showing a sharp resonance near the TO phonon wavelength of 10.2 μm. (b) Dispersion map (9–11 μm) as a function of graphene nanoribbon width $W_{G}$ at fixed chemical potential $E_{F}=0.65\;\text{eV}$, revealing the evolution of thermal emission modes, including the formation of LP, MP, and UP branches. (c) Dispersion tuning with varying chemical potential $E_{F}$ at a fixed $W_{G}=0.15\;\mu m$, demonstrating the reconfigurability of hybrid modes and spectral tuning for mid‐infrared thermal emission. Solid lines in (b) and (c) correspond to theoretical fits from the three‐harmonic oscillator model.

In the graphene–LiV_2_O_5_ subsystem, Figure 6 further explores the interaction between graphene plasmons and phonon modes. As shown in Figure 6a, the emissivity spectrum of a bare LiV_2_O_5_ film clearly reveals a sharp peak at around 10.2 μm, primarily attributed to the local excitation of phonon polaritons along the c‐axis in the RB‐c1 vibrational mode [14]. This resonance closely matches the TO phonon frequency obtained from the dispersion maps in Figure 6b,c. By performing RCWA, we map the hybrid system's dispersion and observe clear anticrossing features between the graphene SPP and the phonon modes of LiV_2_O_5_. At both a graphene width of 0.15 μm and a chemical potential of 0.65 eV, the system exhibits a Rabi splitting of 4.32 meV. This energy separation results in three distinct hybrid modes, namely the LP, MP, and UP branches. We use a three‐harmonic oscillator model that captures the hybrid‐mode dynamics governed by graphene nanoribbon plasmons and phonons [21]:

(10) $$\left[\begin{array}{ccc} \hbar \omega_{\text{SPP}}-i\hbar \frac{\gamma_{\text{SPP}}}{2} & g_{\text{SPP}-\text{TO}} & g_{\text{SPP}-\text{LO}}\\ g_{\text{SPP}-\text{TO}} & \hbar \omega_{\text{TO}}-i\frac{\gamma_{\text{TO}}}{2} & 0\\ g_{\text{SPP}-\text{LO}} & 0 & \hbar \omega_{\text{LO}}-i\frac{\gamma_{\text{LO}}}{2} \end{array}\right]\left[\begin{array}{l} \alpha_{\text{SPP}}\\ \alpha_{\text{TO}}\\ \alpha_{\text{LO}} \end{array}\right]=\hbar \omega \left[\begin{array}{l} \alpha_{\text{SPP}}\\ \alpha_{\text{TO}}\\ \alpha_{\text{LO}} \end{array}\right]$$

where $\gamma_{\text{SPP}}$, $\gamma_{\text{TO}}$, $\gamma_{\text{LO}}$ and $\hbar \omega_{\text{SPP}}$, $\hbar \omega_{\text{TO}}$, $\hbar \omega_{\text{LO}}$ denote the linewidths and resonance energies of the uncouple

SPP, TO, and LO modes, respectively. The off‐diagonal terms $g_{\text{SPP}-\text{TO}}$ and $g_{\text{SPP}-\text{LO}}$ represent the coupling strengths between the SPP and respective phonon modes. These modal fractions are described by eigenvector coefficients ${\left|\alpha_{\text{SPP}}\right|}^{2}$, ${\left|\alpha_{\text{TO}}\right|}^{2}$, ${\left|\alpha_{\text{LO}}\right|}^{2}$, which sum to unity. In Figure 6b, the model captures the dispersion evolution as the graphene width $W_{G}$ varies at a fixed chemical potential $E_{F}=0.65\;\text{eV}$, revealing spectral shifts and mode splitting. Figure 6c further demonstrates consistent agreement under electrostatic tuning of $E_{F}$ at constant $W_{G}=0.15\;\mu m$.

Figure 7 demonstrates reconfigurable multiband thermal emission in the graphene–LiV_2_O_5_ subsystem through complementary structural and electrostatic tuning. Figure 7a shows a pronounced redshift of the LP and UP peaks as the graphene nanoribbon width increases, whereas the MP branch remains comparatively stable near the phonon band. Figure 7b clarifies the origin of this behavior through Hopfield fractions derived from the three‐oscillator model in Equation (10).

**FIGURE 7:**
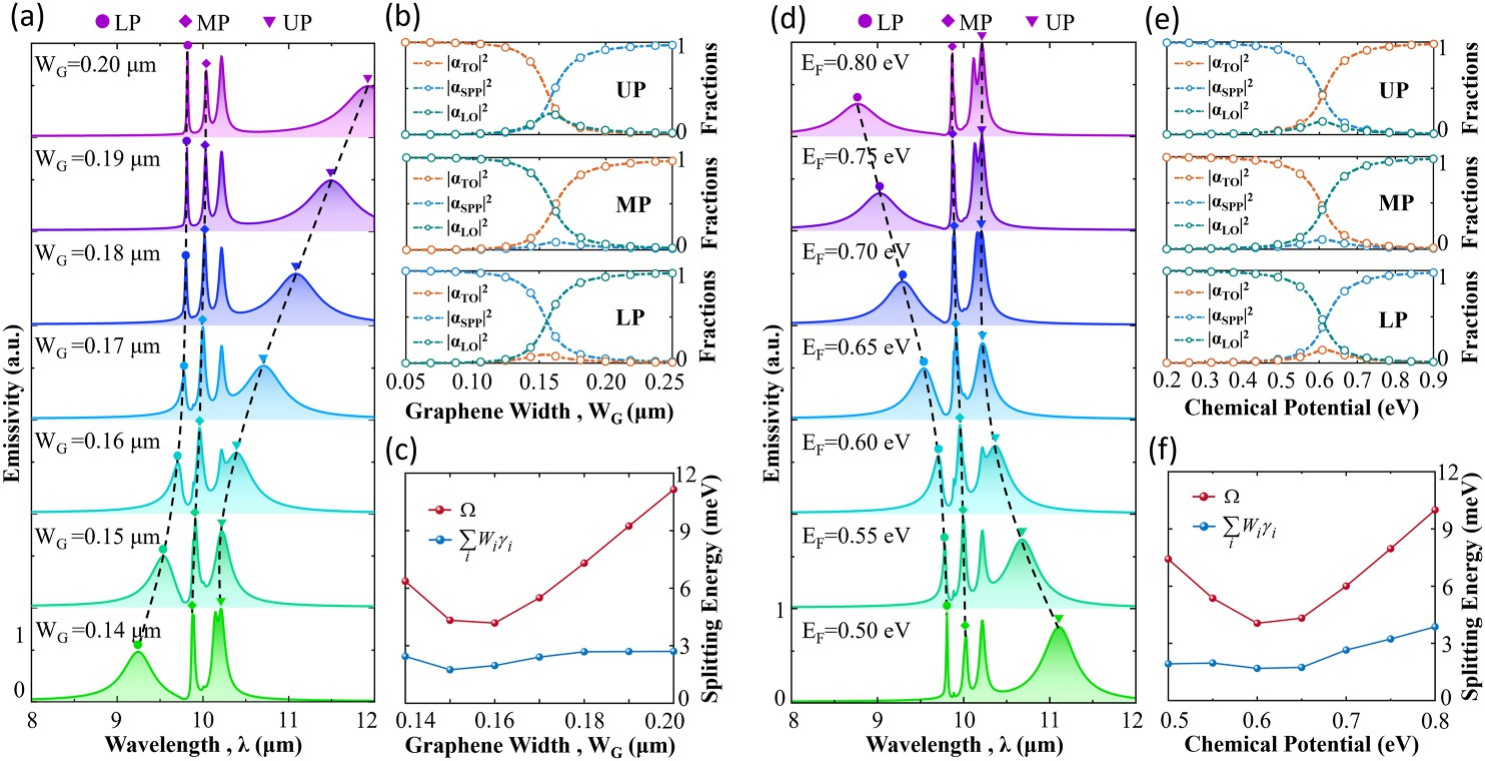
Modal evolution and spectral modulation in the graphene–LiV_2_O_5_ subsystem. (a) Emissivity spectra as a function of graphene nanoribbon width $W_{G}$, showing the emergence of LP, MP, and UP modes near 10 μm. (b) Mode mixing fractions derived from the three‐mode model, revealing the redistribution of energy contributions among SPP, TO, and LO components with increasing $W_{G}$. (c) Extracted spectral splitting energy (Ω) and cumulative linewidth‐weighted modal loss $\sum W_{i}\gamma_{i}$, demonstrating increased spectral tunability with structural control. (d) Emissivity spectra under electrostatic tuning of the graphene chemical potential $E_{F}$, highlighting dynamic shifts in modal positions. (e) Evolution of mixing fractions with $E_{F}$, reflecting carrier‐controlled energy redistribution and mode reconfiguration. (f) Nonmonotonic variation of Ω and $\sum W_{i}\gamma_{i}$ with $E_{F}$, indicating reconfigurable dual‐band thermal emission under electronic control.

With increasing ribbon width, the LP branch evolves from SPP‐dominated to phonon‐dominated character with a dominant LO contribution. The MP branch remains largely phononic with only weak SPP weight, whereas the UP branch becomes increasingly plasmonic and reduces its TO participation. Figure 7c quantifies the corresponding coherent coupling by tracking the Rabi splitting energy and the cumulative linewidth‐weighted loss. The threshold criterion follows the standard non‐Hermitian three‐mode coupled‐oscillator formalism and confirms that the observed splittings arise from coherent mode mixing rather than accidental spectral overlap, thereby distinguishing genuine hybridization from simple resonance crossing [43, 44, 45].

Specifically, the coupling condition is expressed as a threshold criterion:

(11) $$\Omega >W_{\text{UP}}\gamma_{\text{UP}}+W_{\text{MP}}\gamma_{\text{MP}}+W_{\text{LP}}\gamma_{\text{LP}}$$

Here, Ω denotes the Rabi splitting energy, whereas $\gamma_{\text{UP}}$, $\gamma_{\text{MP}}$, and $\gamma_{\text{LP}}$ correspond to the linewidths of the UP, MP, and LP branches, respectively. $W_{\text{UP}}$, $W_{\text{MP}}$ and $W_{\text{LP}}$ represent the fractional contributions in each hybrid mode, obtained through eigenvector decomposition of the system's Hamiltonian. Strong coupling is set by the balance between coherent exchange and dissipative loss. This three‐mode form is required because the graphene SPP is simultaneously coupled to the TO and LO phonons of LiV_2_O_5_. The criterion compares the effective coupling strengths with the corresponding loss rates and incorporates branch‐dependent mixing weights, thereby capturing unequal coupling strengths and loss asymmetry among the three bare modes [43, 44]. The analytical expressions for $W_{\text{UP}}$, $W_{\text{MP}}$ and $W_{\text{LP}}$ can be expressed as follows:

(12) $$\left\{\begin{array}{l} W_{\text{UP}}=\frac{\gamma_{\text{UP}}}{\gamma_{\text{UP}}+\gamma_{\text{MP}}+\gamma_{\text{LP}}}\\ W_{\text{MP}}=\frac{\gamma_{\text{MP}}}{\gamma_{\text{UP}}+\gamma_{\text{MP}}+\gamma_{\text{LP}}}\\ W_{\text{LP}}=\frac{\gamma_{\text{LP}}}{\gamma_{\text{UP}}+\gamma_{\text{MP}}+\gamma_{\text{LP}}} \end{array}\right.$$

Furthermore, we analyze the modal composition and linewidth evolution based on extracted Hopfield mixing fractions. Under representative conditions $W_{G}=0.15\;\mu m$ and $E_{F}=0.65\;\text{eV}$, the mode‐specific linewidths are expressed as:

(13) $$\left[\begin{array}{l} \gamma_{\text{UP}}\\ \gamma_{\text{MP}}\\ \gamma_{\text{LP}} \end{array}\right]=\left[\begin{array}{ccc} 17.27\% & 73.51\% & 9.22\%\\ 7.64\% & 20.15\% & 72.21\%\\ 70.64\% & 10.52\% & 18.84\% \end{array}\right]\left[\begin{array}{l} \gamma_{\text{SPP}}\\ \gamma_{\text{TO}}\\ \gamma_{\text{LO}} \end{array}\right]$$

Given the linewidths $\gamma_{\text{SPP}}=3.25\;\text{meV}$ and $\gamma_{\text{TO}}=\gamma_{\text{LO}}=0.48\;\text{meV}$, we calculate $\gamma_{\text{UP}}=0.96\;\text{meV}$, $\gamma_{\text{MP}}=0.69\;\text{meV}$, and $\gamma_{\text{LP}}=2.44\;\text{meV}$. These values yield corresponding Hopfield weights of $W_{\text{UP}}=0.235$, $W_{\text{MP}}=0.169$, and $W_{\text{LP}}=0.596$. Substituting these values into the multi‐mode threshold criterion in Equation (11), we obtain a minimum Rabi splitting of:

(14) $$\Omega >0.235\gamma_{\text{UP}}+0.169\gamma_{\text{MP}}+0.596\gamma_{\text{LP}}$$

This criterion reflects a physical balance between coupling‐driven energy exchange and total system loss. Unlike simpler detuning‐based estimators, it accounts for both loss asymmetry and unequal coupling strengths across modes. The Rabi splitting extracted from the dispersion relation (Ω=4.32meV) exceeds this threshold, confirming the system's entry into the hybridization regime. Figure 7d shows the complementary response under electrostatic control. Increasing the Fermi level blueshifts LP and UP, consistent with plasmon stiffening. Figure 7e reveals a reversed redistribution of mode composition. Higher Fermi level increases the SPP fraction in LP, whereas UP becomes more phonon‐like, enabling continuous steering of the multiband emission landscape. Figure 7f further shows a nonmonotonic evolution of the Rabi splitting energy and linewidth‐weighted loss with gating, underscoring reconfigurable hybridization under electronic control. The resulting branch set remains narrowband and functionally reconfigurable. The MP branch preserves high spectral selectivity with a representative quality factor of 138.97. The LP branch shows the largest emissivity redistribution, and its response switches with the tuning parameter. Among the long‐wavelength branches, LP_2_ shows the largest emissivity redistribution, and its response switches with the tuning parameter. Under geometric modulation, LP_2_ is strongly enhanced, with $C_{\varepsilon }=0.70$ and $R_{\varepsilon }=1.70$. Under electrostatic gating, the same branch is strongly suppressed, with $C_{\varepsilon }=-0.64$ and $R_{\varepsilon }=0.36$. LP_2_ also reaches $FOM_{\lambda }$ up to 49.33, indicating a large tuning span relative to its linewidth. Full statistics for Q, contrast, and $FOM_{\lambda }$ are listed in Supporting Information S1: Tables S2 and S3, and benchmarking against representative emitters is summarized in Supporting Information S1: Table S4.

## 4 Fabrication Feasibility and Process Considerations

The proposed graphene–2D‐hBN–LiV_2_O_5_ heterostructure is compatible with established van der Waals assembly and mid‐infrared nanofabrication techniques. LiV_2_O_5_ can be incorporated a continuous and unpatterned phonon‐active interlayer, since its infrared response is governed by bulk anisotropic phonon modes rather than lateral resonator geometry. Thickness‐controlled LiV_2_O_5_ films can be obtained from flux‐grown crystals by mechanical or polymer‐assisted exfoliation, and recent studies have shown that such films retain clear Reststrahlen‐band features and low‐loss phonon‐polariton responses on dielectric or metallic substrates [46, 47, 48]. Moderate Nanoscale thickness nonuniformity is therefore expected mainly to broaden the long‐wavelength resonance, without suppressing the LiV_2_O_5_‐mediated modulation channel.

The graphene–2D‐hBN stack can be assembled on the BaF_2_/LiV_2_O_5_/BaF_2_/Ag platform using dry‐transfer or polymer‐assisted transfer methods. hBN serves both as the short‐wavelength phononic medium and as an atomically flat buffer for graphene, helping to preserve mobility and reduce substrate‐induced disorder [15, 16, 49, 50]. Graphene nanoribbons with submicrometer linewidths can then be patterned by electron‐beam lithography or nanoimprint lithography, which are compatible with CVD‐grown or exfoliated graphene [51]. Electrostatic tuning can be implemented using bottom‐gate or dual‐gate architectures; in this design, the Ag reflector can serve as a global bottom electrode, whereas BaF_2_ acts as a low‐loss infrared spacer and gate dielectric [50, 52, 53, 54].

The predicted hybridization can be verified by tracking gate‐ and geometry‐dependent far‐field spectra. Since the Ag reflector suppresses transmission, the emissivity can be extracted from reflectance through Kirchhoff's law, ε(λ)=1−R(λ), using FTIR‐based measurements [24, 55]. Split peaks near the hBN phonon band and multibranch features within the LiV_2_O_5_ Reststrahlen band would provide direct evidence of avoided crossings and plasmon–phonon hybridization. Their systematic shifts with graphene Fermi level and nanoribbon width would further confirm dynamic, reversible dual‐band thermal‐emission modulation.

The main practical challenges are maintaining graphene mobility, minimizing transfer residues and interface contamination, and preserving LiV_2_O_5_ crystallinity and thickness uniformity. These issues can be addressed by hBN encapsulation, clean transfer, post‐transfer annealing and optical preselection of uniform LiV_2_O_5_ flakes [15, 16, 46, 47, 48, 49, 50]. Residual imperfections may broaden resonances or reduce modulation depth, but they should not alter the plasmon–phonon coupling mechanism underlying the proposed dual‐band emitter [53, 54, 55].

## 5 Conclusion

We demonstrate a dual‐band tunable thermal emission platform based on a graphene–2D‐hBN–LiV_2_O_5_ heterostructure for dynamic control of mid‐infrared radiation. RCWA calculations combined with coupled‐oscillator modeling reveal spectrally tunable emission near 7 and 10 μm enabled by band‐selective plasmon–phonon hybridization. Low‐loss TO phonons and hyperbolic dispersion in 2D‐hBN underpin narrowband coherent emission around 7 μm, whereas broadband anisotropic phonons in LiV_2_O_5_ support multimode interactions that yield tunable emission near 10 μm. Independent tuning of nanoribbon width and graphene Fermi level provides dynamic control over both emissivity and resonance wavelength, with LP and UP branches exhibiting opposite shifts that facilitate mode‐resolved emission shaping. Multilayer 2D‐hBN further enhances confinement and emission efficiency. The resulting non‐monotonic spectral evolution is captured by a multimode coupling criterion, demonstrating robust and flexible dual‐band operation. These findings outline a general design strategy for reconfigurable mid‐infrared thermal emitters and support radiative control in nanophotonic systems.

## Author Contributions

**Yuhui Cai:** conceptualization, investigation, writing – original draft, methodology, validation, visualization, writing – review and editing, software, formal analysis, project administration, data curation. **Minru Zhu:** conceptualization, investigation, writing – original draft, writing – review and editing, visualization, validation, methodology, software, formal analysis, project administration, data curation. **Peiyuan Huang:** writing – review and editing, project administration, validation, investigation, conceptualization. **Qinmiao Chen:** writing – review and editing, project administration, supervision, formal analysis. **Xuening Wang:** investigation, supervision, formal analysis, writing – review and editing. **Bingfeng Fan:** writing – review and editing, project administration, supervision, resources. **Yongyao Li:** writing – review and editing, project administration, resources, supervision. **Jiayao Huang:** funding acquisition, writing – review and editing, project administration, resources, supervision, formal analysis, investigation. **Dongxu Zhao:** project administration, resources, supervision, writing – review and editing.

## Conflicts of Interest

The authors declare no conflicts of interest.

## Supporting information


Supporting Information S1

## Data Availability

The datasets generated and/or analyzed during the current study are available from the corresponding author upon reasonable request.
