# Supplementary material for: Dynamical Modulation of Dual‐Band Thermal Emission in a Graphene‐2D‐hBN‐LiV2O5 Heterostructure
Source: Nanophotonics. 2026 Jun 28;15(13):e70180. doi: 10.1002/nap2.70180 (PMC13642611; doi:10.1002/nap2.70180)
Supplement: Supplementary file 1 [file NAP2-15-e70180-s001.docx]

Supplementary Information

**Dynamical modulation of dual-band thermal emission in a Graphene–2D-hBN–LiV_2_O_5_ Heterostructure**

Yuhui Cai, Minru Zhu, Peiyuan Huang, Qinmiao Chen, Xuening Wang, Bingfeng Fan, Yongyao Li, Jiayao Huang*, Dongxu Zhao*

*Yuhui Cai and Minru Zhu contributed equally to this work.*

**Corresponding authors: Jiayao Huang and Dongxu Zhao, School of Physics and Optoelectronic Engineering, Foshan University, Foshan, Guangdong 528225, China,*

*Email: huangjiayao@fosu.edu.cn (J. Huang),*

*Email: zhaodx@fosu.edu.cn (D. Zhao).*

*Yuhui Cai, Minru Zhu, Peiyuan Huang, Xuening Wang, Bingfeng Fan and Yongyao Li, School of Physics and Optoelectronic Engineering, Foshan University, Foshan, Guangdong 528225, China.*

*Qinmiao Chen, Department of Physics, The Hong Kong University of Science and Technology, Clear Water Bay, 999077 Kowloon, Hong Kong, China.*

**Section I: Dual-channel independence verification**

Figure S1 shows that the dual-band emission in the graphene–2D-hBN–LiV₂O₅ heterostructure arises from two spectrally independent channels. Figures S1(a) and (d) first isolate the graphene response under nanoribbon width tuning and chemical potential tuning. In both cases, the graphene SPP branch evolves continuously across the mid-infrared range, confirming that graphene provides the tunable plasmonic mode for both geometric and electrostatic modulation. Figures S1(b) and (e) consider the phononic stack without graphene and reveal two distinct resonances near 7 μm and 10 μm. These resonances indicate that hBN and LiV₂O₅ each sustain a separate phonon channel associated with their TO phonons, without observable interlayer hybridization.

When all layers are combined, Figure S1(c) shows that the width tuning of graphene produces two spectrally isolated avoided crossings near the hBN and LiV₂O₅ phonon resonances. Figure S1(f) further shows that the chemical potential tuning leads to the same channel-selective hybridization, with the gate-tuned graphene SPP selectively coupling to the fixed phonon resonances of LiV₂O₅ and hBN. The resulting LP₁/UP₁ and LP₂/MP₂/UP₂ branches remain spectrally separated, forming two independent hybrid-polariton manifolds in the short- and long-wavelength bands. This spectral separation provides the basis for dual-band thermal-emission modulation in the heterostructure.

**Figure S1:** Spectral independence of the two emission channels in the graphene–2D-hBN–LiV₂O₅ heterostructure.

**Section II: LiV₂O₅ anisotropy and orientation dependence**

LiV₂O₅ exhibits strong optical anisotropy, which controls the strength and spectral definition of its Reststrahlen response in the long-wavelength band. Figures S2(a) and (b) show that the a-axis and c-axis dielectric functions display pronounced phonon resonances, evidenced by negative real permittivity and strong absorption peaks, whereas the b-axis response remains weak over the same range. This anisotropy motivates our representative choice in the main text, where the c-axis response is used because it yields a strong and spectroscopically well-defined Reststrahlen channel around 10 μm and therefore provides a clean long-wavelength emission band for the dual-band architecture. The dual-band mechanism remains robust with respect to orientation. Figure S2(c) compares normalized emissivity spectra for a-axis and c-axis cases and confirms the persistence of the long-wavelength hybrid features. Figures S2(d) and (e) further show emissivity maps versus graphene width at *E*_F_ equal to 0.65 eV. In both orientations the hybrid branches LP_2_ MP_2_ and UP_2_ appear in the 9–11 μm window and shift systematically with graphene width. Orientation mainly reshapes relative peak amplitudes and linewidth, while the existence and tunability of the long-wavelength hybrid polaritons remain unchanged.

**Figure S2:** Anisotropic dielectric response of LiV₂O₅ and its influence on dual-band hybrid-polariton emission in the graphene–2D-hBN–LiV₂O₅ heterostructure.

**Section III: Interlayer phonon–phonon interaction between hBN and LiV₂O₅**

We evaluate whether the hBN and LiV₂O₅ phonon responses remain spectrally independent when stacked directly, since an hBN overlayer could in principle perturb LiV₂O₅ phonon polaritons through dielectric-environment effects. We perform graphene-free RCWA control simulations of the hBN–LiV₂O₅ stack to identify any intrinsic phonon–phonon hybridization or spectral crosstalk. Figure S3(a) varies the hBN thickness from one to five layers while keeping LiV₂O₅ at 30 nm. Figure S3(b) varies the LiV₂O₅ thickness from 10 to 50 nm while keeping hBN at one layer. Both parameter sweeps preserve two distinct resonances near 7 μm and 10 μm. The 7 μm feature responds primarily to the hBN thickness, whereas the 10 μm feature responds primarily to the LiV₂O₅ thickness. The peak positions remain well separated and no mode repulsion or mixed lineshape develops across the tuning range. These results show that the stack does not enter a strong phonon–phonon coupling regime under the considered conditions. Instead, hBN provides only weak dielectric loading of the LiV₂O₅ channel. The large detuning between their Reststrahlen bands suppresses coherent interlayer hybridization and ensures negligible crosstalk, which supports the independent dual-band design strategy used in the full heterostructure.

**Figure S3:** Dynamic dual-band thermal-emission modulation in the 2D-hBN–LiV₂O₅ heterostructure.

**Section IV: Angular and polarization dependence of dual-band thermal emission**

The ENZ and Berreman type modes can couple to cavity, plasmonic or polaritonic resonances in suitable geometries [1–5]. To separate these effects from the plasmon phonon hybridization discussed in the main text, we calculated angular and polarization resolved emissivity spectra for the hBN and LiV₂O₅ channels, as shown in Fig. S4. A Berreman type response is typically activated by the normal electric field under TM polarized oblique incidence and is strongly suppressed for TE polarization or near normal incidence [1, 3, 4]. For the hBN channel, the 7.2 μm peak is present at normal incidence and decreases as the incident angle increases from 0° to 80°, as shown in Fig. S4(a). At $\theta={80}^{\circ}$, the TE response is stronger than the TM response, as shown in Fig. S4(c). This behaviour is inconsistent with an hBN ENZ or Berreman type origin and instead identifies the feature as the infrared active hBN TO phonon. The LP₁/UP₁ splitting therefore arises from graphene SPP coupling to the hBN phonon resonance. For LiV₂O₅, an ENZ related feature appears under large angle TM excitation, as shown in Figs. S4(b) and (d). The inset of Fig. S4(d) shows that Re(ε) approaches zero at λ_ENZ_ = 9.76 μm, consistent with the narrow TM selective feature near this wavelength. This response is absent for TE polarization and is spectrally separated from the dominant LiV₂O₅ TO phonon emission near 10.2 μm. It contributes mainly to oblique incidence angular emissivity, rather than to the LP₂/MP₂/UP₂ splitting analysed under normal incidence. Consequently, the dual band hybrid branches in the main text are attributed to graphene SPP coupling with hBN and LiV₂O₅ phonons. Their anti-crossing behaviour and Rabi splittings follow the graphene nanoribbon width and Fermi level, and are reproduced by the coupled oscillator models. ENZ effects in LiV₂O₅ are therefore treated as a secondary angular emission contribution, not the origin of the normal incidence plasmon phonon splitting.

**Figure S4:** Angular and polarization characteristics of emissivity in the hBN and LiV₂O₅ channels. (a) Waterfall emissivity spectra of hBN at different incident angles. (b) Waterfall emissivity spectra of LiV₂O₅ at different incident angles. (c) TE/TM comparison of hBN at $\theta={80}^{\circ}$. (d) TE/TM comparison of LiV₂O₅ at $\theta={80}^{\circ}$.

To clarify the influence of ENZ effects on the angular thermal emission in Fig. S5, we calculated TM polarized emissivity spectra for the graphene–hBN and graphene–LiV₂O₅ channels from 0° to 80°. The waterfall representation separates the angular evolution of each resonance and allows the ENZ related response to be distinguished from the main plasmon–phonon hybrid branches. For the graphene–hBN channel, the LP and UP resonances near 7 μm remain nearly fixed in wavelength as the incident angle increases. The angular dependence is mainly reflected in the variation of peak intensity, rather than in a pronounced spectral shift. This indicates that the short wavelength channel is dominated by graphene SPP coupling to the hBN phonon resonance, and that ENZ effects do not play a primary role in the hBN related splitting. The graphene–LiV₂O₅ channel shows a stronger angular dependence. In addition to intensity variations, the LP, MP and UP features exhibit moderate wavelength shifts under oblique TM incidence. This behaviour is consistent with the ENZ response of LiV₂O₅ in this spectral region. Under oblique TM excitation, the normal electric field component can couple to the ENZ related response, which modifies the angular emissivity background and slightly perturbs the apparent positions of the graphene–LiV₂O₅ polaritonic peaks. Therefore, the ENZ contribution is angle dependent and mainly affects the oblique incidence emissivity of the LiV₂O₅ channel. It does not change the interpretation of the normal incidence spectra discussed in the main text, where the LP₂, MP₂ and UP₂ branches are governed by tunable graphene SPP coupling to the LiV₂O₅ TO and LO phonons. ENZ effects are thus treated as a secondary angular emission contribution rather than the origin of the dual band plasmon–phonon splitting in the graphene–2D-hBN–LiV₂O₅ emitter.

**Figure S5:** TM-polarized angle-dependent emissivity spectra for θ from 0° to 80° in the (a) graphene–hBN channel and the (b) graphene–LiV₂O₅ channel.

**Section V: Temperature-weighted emission spectrum**

We evaluate thermal emission using a Planck-weighted description that accounts for temperature-dependent radiative exitance. The spectral emissivity is defined as the ratio between the radiative exitance of the heterostructure and that of an ideal blackbody at the same temperature, expressed as:

 (S1)

where $M_{\text{structure}}$ and $M_{\text{BB}}$ denote the radiative exitance of the heterostructure and the blackbody at the same temperature, respectively. The latter follows Planck's law:

$M_{\text{BB}}\left(\lambda\right)\text{=}\frac{\text{2π}h\text{c}}{\lambda^{5}\left(\text{e}^{\frac{h\text{c}}{\lambda k\text{T}}}\text{-1}\right)}$ (S2)

Figures S6(a) and (b) show that both channels exhibit strong temperature robustness over 100–500 K. The resonance wavelengths remain nearly constant, with graphene–hBN peaks at 6.7 μm for LP_1_ and 7.4 μm for UP_1_, and graphene–LiV₂O₅ peaks at 9.5 μm for LP_2_, 10.0 μm for MP_2_, and 10.2 μm for UP_2_ across 100–500 K. These observations confirms that operating temperature is not the primary parameter governing the resonance frequency. The behaviour is expected because the eigenfrequency of the dominant plasmon–phonon polariton mode is mainly determined by graphene Fermi level and geometric electromagnetic coupling, and is only weakly temperature dependent [6]. In contrast, the peak radiative exitance increases markedly with temperature within the characteristic bands. The hBN-channel LP rises from 31.053 to 93.763 W·m⁻²·μm⁻¹ when heating from 400 K to 500 K, and the LiV₂O₅-channel LP increases from 19.442 to 42.468 W·m⁻²·μm⁻¹ over the same interval, consistent with the overall increase predicted by Planck radiation. The emission peaks also show nearly temperature-invariant linewidths across 100–500 K. For example, the full width at half maximum of the LiV₂O₅-channel LP remains on the order of 0.12 μm across 100–500 K, indicating that temperature is not the dominant factor controlling spectral broadening. This stability is consistent with slowly varying intrinsic optical-phonon linewidths in high-quality single-crystal hBN [7] and with strong-coupling-induced hybridization that can reduce temperature-sensitive scattering so that radiative or interface-related losses dominate the observed linewidth [8]. A direct comparison with the blackbody reference in Figs. S6(c) and (d) further highlights the advantage of controllable dual-band narrowband thermal emission. In the same mid-infrared windows, the blackbody reference exhibits a smooth broadband continuum that reduces spectral radiance within any prescribed narrow window. By contrast, the graphene–hBN and graphene–LiV₂O₅ channels concentrate emission into designed bands of approximately 6–8 μm and 9–11 μm, respectively.

**Figure S6:** Temperature-dependent radiant exitance of the graphene–hBN channel at 5–9 μm and the graphene–LiV₂O₅ channel at 8–12 μm, with blackbody references.

Figure S7 establishes that the heterostructure behaves as a dual-band narrowband thermal emitter with near-blackbody peak performance. Figure S7(a) shows that the graphene–hBN channel produces narrow features in the 6–8 μm window whose peak radiative exitance nearly matches the blackbody curve at 300 K, while the off-resonant background remains strongly suppressed. Figure S7(b) shows the same behaviour for the graphene–LiV₂O₅ channel in the 9–11 μm window. The combination of near-unity peaks and low off-resonant emission demonstrates spectrally selective dual-band radiation rather than broadband emission. The origin of these peaks is clarified by the layer-resolved emissivity in Figs. S7(c) and (d). The constituent-only spectra show isolated graphene SPP and phonon resonances, whereas the coupled channels exhibit hybrid polaritonic branches. In the graphene–hBN channel the LP and UP peaks emerge only in the coupled configuration, and in the graphene–LiV₂O₅ channel the coupled response further develops an additional MP branch alongside LP and UP. The coupled structure also sustains higher peak emissivity than the constituent baselines at the same wavelengths. The peak emissivity reaches 0.96 in the graphene–hBN channel, compared with 0.63 for graphene-only and 0.39 for hBN-only. It reaches 0.94 in the graphene–LiV₂O₅ channel, compared with 0.49 for graphene-only and 0.93 for LiV₂O₅-only. Together, these results link the near-blackbody peaks to plasmon–phonon hybridization and its associated field confinement in the designed spectral windows.

**Figure S7:** Blackbody-referenced emission and layer-resolved emissivity of the graphene–hBN and graphene–LiV₂O₅ channels at 300 K.

**Section VI: Comparison with other dual-band thermal emitters**

Table S1 benchmarks representative dual-band and multi-band thermal emitters and highlights a recurring trade-off between spectral selectivity and tunability. Geometry-coded metasurfaces and multilayer resonators can deliver narrowband dual features that are spatially distinguishable or mode selective, but their operating wavelengths are largely set by the fabricated dimensions and remain fixed after fabrication [9, 10]. In these platforms, tunability is primarily achieved through geometric variation across different devices rather than continuous reconfiguration within a single device, consistent with the entries in Table S1. A distinct class employs phase-change materials and insulator-to-metal-transition media to switch between emission states. These devices can yield pronounced spectral contrast and large inter-state shifts through thermal switching, but the modulation typically proceeds through discrete states and does not provide continuous analog control of resonance positions within a given band. This behaviour reflects the switching-based mechanisms summarized in Table S1.

The present work adopts a different paradigm. Dual-band emission arises from hybridization-defined polaritonic branches in a single graphene–2D-hBN–LiV₂O₅ platform rather than from juxtaposed independent resonances. The two channels remain spectrally separated and each hosts multiple hybrid branches. Electrostatic tuning of graphene together with geometric control of the nanoribbon width enables continuous and reversible programming of both bands within one device, supporting tunable operation across two mid-infrared atmospheric windows while retaining narrowband polaritonic selectivity, as captured by the final row in Table S1.

**Table S1:** Comparative Summary of Recent Dual-/Multi-Band Thermal Emitters

| **Work & platform** | **Spectral bands (****λ_1_,** **λ_2_)** | **Spectral selectivity / coherence** | **Tunability mechanism** |
| --- | --- | --- | --- |
| Dual-band polaritonic nanoresonator [9] | 6.62 µm & 6.56 µm | Narrowband & spatially distinguishable (near-field-resolved) | **Geometry** tuning |
| Au/Al₂O₃/Au MIM metasurface thermal emitter [10] | 4.37 µm & 2.27 µm | Narrowband & spatially distinguishable (angle-separated MPs) | **Geometry** tuning |
| Static dual-band MIM plasmonic absorber (Al-Al₂O₃-Al) [11] | 4.16 μm & 8.65 μm | Narrowband (M1: SP–photonic coupling; M2: magnetic resonance) | **Geometry** tuning |
| Dual-band switchable metasurface emitter (GST nanodisks) [12] | a-GST: 5.4, 7.4 µm  c-GST: 7.6, 10 µm | Narrowband & switchable dual-resonant emission | Thermal switching (discrete PCM) |
| VO₂ microdisk metasurface thermal emitter (VO₂/HfO₂/Al) [13] | Insulating VO₂: ~3 μm; Metallic VO₂: ~5 μm | Narrowband (Magnetic polaritons) | Thermal switching (via IMT) |
| Polarization-switchable MIM plasmonic thermal emitter (GST + Au ellipses) [14] | a-GST: 8.3, 9.6 μm  40% c-GST: 9.3, 10.6 μm | Narrowband & switchable dual-resonant emission | Thermal switching (discrete PCM) |
| **This work: Graphene–2D-hBN–LiV₂O₅ heterostructure** | **~7 μm + ~10 μm (atmospheric windows)** | **Hybridization-defined narrowband polaritonic channels; five branches (LP₁/UP₁ and LP₂/MP₂/UP₂)** | **Continuous electrostatic tuning (*E*_F_) + geometry (*W*_G_)** |

**Section VII: Eigenvalues of the two-harmonic oscillator model**

For the graphene–2D-hBN system, the complex eigenvalues obtained by diagonalizing the Hamiltonian in Eq. (6) are

 （S3）

The eigenvalues obtained from Eq. (S3) are shown as solid dots in Figs. 3(b) and (c), exhibiting excellent agreement with the RCWA-derived dispersion relations and confirming the descriptive accuracy of the coupled oscillator model.

**Section VIII: Plasmon polariton and phonon polariton ribbon resonances**

Quantitative evaluation of reconfigurable dual-band thermal emission requires metrics that capture linewidth, intensity programmability, and tuning span. We use the quality factor ($Q$) with $Q=\lambda_{0}/\Delta\lambda_{\text{FWHM}}$ to describe narrowband selectivity [15]. We use modulation contrast $C_{\text{ε}}$ and ratio $R_{\text{ε}}$ to quantify reversible intensity reallocation through $C_{\varepsilon}\left(\lambda_{0}\right)=[\varepsilon_{\text{on}}\left(\lambda_{0}\right)-\varepsilon_{\text{off}}\left(\lambda_{0}\right)]/\varepsilon_{\text{off}}$ and $R_{\varepsilon}\left(\lambda_{0}\right)=\varepsilon_{\text{on}}\left(\lambda_{0}\right)/\varepsilon_{\text{off}}\left(\lambda_{0}\right)$ [16, 17]. The on and off states are defined by the specific configurations in Figs. 5 and 7. We further use figures of merit $FOM_{\text{λ}}$, expressed as $FOM_{\text{λ}}=\Delta\lambda_{\text{tune}}/\Delta\lambda_{\text{FWHM}}$ [18]. It is defined as tuning range normalized by the linewidth, to assess whether the tuning is practically meaningful relative to the intrinsic spectral resolution.

Most reported dual-band emitters do not provide systematic Q-factors, contrast, and figures of merit for continuous and reversible reconfiguration. Static metal–insulator–metal approaches such as Au/Al_2_O_3_/Au metasurface emitters set two resonances by design and offer no active control beyond geometry. Phase-change metasurfaces such as GST nanodisk designs rely on discrete switching and are limited by thermal hysteresis and cycling endurance, limiting fine reversible tuning [9, 10].

Tables S2 and S3 summarize these metrics for all hybrid branches under geometric modulation and electrostatic control. Across both channels, the hybrid modes exhibit Q-factors in the range 29–139. The emissivity modulation is strongly branch selective rather than a uniform scaling of intensity. Under geometric modulation, LP_2_ shows a large enhancement with $C_{\text{ε}}$ equal to 0.70 and $R_{\text{ε}}$ equal to 1.70, while UP_1_ is suppressed with $C_{\text{ε}}$ equal to −0.25 and $R_{\text{ε}}$ equal to 0.75, and UP_2_ is suppressed with $C_{\text{ε}}$ equal to −0.45 and $R_{\text{ε}}$ equal to 0.55. Under electrostatic control, LP_2_ instead becomes strongly suppressed with $C_{\text{ε}}$ equal to −0.64 and $R_{\text{ε}}$ equal to 0.36, while other branches can be enhanced, for example LP_1_ with $C_{\text{ε}}$ equal to 0.18 and $R_{\text{ε}}$ equal to 1.18, and UP_2_ with $C_{\text{ε}}$ equal to 0.23 and $R_{\text{ε}}$ equal to 1.23. The figures of merit spans $FOM_{\text{λ}}$ equal to 1.33–49.33, which indicates tuning over one to several tens of linewidths without sacrificing narrowband character.

Table S4 benchmarks this performance against representative narrowband emitters including a quasi-BIC radiator with $Q$ about 42 [19], a VO_2_–GST tunable narrowband emitter based on thermal switching and material-state tuning [20], and a hybrid metal–OTS emitter with $Q$ equal to 780 and $FOM_{\text{λ}}$ equal to 4.6 [21]. The present platform combines a comparable $Q$ range with two spectrally separated channels and continuous reversible multi-branch programmability, which together meet the requirements of a reconfigurable dual-band thermal emitter.

**Table S2:** Relevant Indicators and Parameters under Geometric Modulation

| **Hybrid branches** | $Q$ | $C_{\text{ε}}$ | $R_{\text{ε}}$ | $FOM_{\text{λ}}$ |
| --- | --- | --- | --- | --- |
| UP_1_ | 95.78 | -0.25 | 0.75 | [1.95, 10.29] |
| LP_1_ | 49.51 | 0.05 | 1.05 | [4.42, 6.63] |
| UP_2_ | 45.59 | -0.45 | 0.55 | [2.52, 10.19] |
| MP_2_ | 138.97 | -0.25 | 0.75 | [1.67, 3.33] |
| LP_2_ | 29.06 | 0.70 | 1.70 | [1.33, 31.22] |

**Table S3:** Relevant indicators and parameters under electrostatic control

| **Hybrid branches** | $Q$ | $C_{\text{ε}}$ | $R_{\text{ε}}$ | $FOM_{\text{λ}}$ |
| --- | --- | --- | --- | --- |
| UP_1_ | 95.78 | 0.06 | 1.06 | [3.32, 13.29] |
| LP_1_ | 49.50 | 0.18 | 1.18 | [4.93, 7.67] |
| UP_2_ | 45.59 | 0.23 | 1.23 | [1.97, 8.54] |
| MP_2_ | 138.97 | 0.20 | 1.20 | [2.33, 4.75] |
| LP_2_ | 29.061 | -0.64 | 0.36 | [1.76, 49.33] |

**Table S4:** Comparison of key metrics for representative narrowband thermal emitters

| **Emitter Design** | **Spectral peaks (µm)** | **Tunability mechanism** | $Q$ | $FOM_{\text{λ}}$ |
| --- | --- | --- | --- | --- |
| **This work** | **~7 µm & ~10 µm** | **electrostatic + geometric** | **29–139** | **1.33–49.33** |
| Au/Al₂O₃/Au MIM metasurface thermal emitter [9] | 4.37 µm & 2.27 µm | **Geometry** tuning | NR | NR |
| Dual-band switchable metasurface emitter (GST nanodisks) [10] | a-GST: ~5.4, ~7.4 µm  c-GST: ~7.6, ~10 µm | Thermal switching (discrete PCM) | NR | NR |
| Quasi-BIC-enabled narrowband thermal radiator [19] | ~4.5 μm | Geometric tuning | ~42 | NR |
| VO₂–GST smart mid-infrared tunable narrowband absorber/thermal emitter [20] | 4.1 µm | Thermal switching + material-state tuning | NR | 1 |
| Hybrid metal–OTS (optical Tamm state) thermal emitter [21] | 4.565 μm | Thermal tuning | 780 | 4.6 |

**Section IX: Mixing fractions in the graphene–2D-hBN two-mode system**

For the two-mode graphene–2D-hBN coupled system, the SPP and TO mixing fractions of the hybrid modes are obtained from the eigenvectors of the coupled Hamiltonian. The corresponding weight factors are:

 (S4)

 (S5)

**Section X: Comparison of hBN dielectric models and modal-fraction analysis**

In this work, the dielectric response of hBN with different layer numbers is described using a layer-dependent modeling scheme. For monolayer hBN, we use the monolayer model described by Eqs. (1) and (2). For $\text{N}\text{ }\text{=}\text{ }\text{2}\text{ to }\text{4}$, hBN is treated as an intermediate quasi-bulk system. The calculation is performed within a three-dimensional anisotropic thin-film framework, with dielectric constants taken from the reported layer-dependent experimental values for the corresponding $\text{N}$-layer hBN [22]. For *N* ≥ 5, the dielectric constants are nearly saturated, and a unified bulk hBN model is therefore adopted.

The corresponding dielectric-function parameters used for different hBN layer numbers are summarized in Table S5:

**Table S5:** Layer-dependent variation of the macroscopic dielectric constant of hBN [22]

| Number of layers | $\varepsilon_{\infty,\perp}$ | $\varepsilon_{\infty,\parallel}$ |
| --- | --- | --- |
| 1 | 4.97 | 2.89 |
| 2 | 4.97 | 2.91 |
| 3 | 4.97 | 2.96 |
| 4 | 4.96 | 2.97 |
| Bulk | 4.98 | 3.03 |

Once hBN becomes sufficiently thick to behave quasi-bulk in the out-of-plane direction, we use the standard LO–TO form [23, 24]:

$\varepsilon_{\text{hBN,}\psi}=\varepsilon_{\infty,\psi}\left(1+\frac{\omega_{\text{LO,}\psi}^{2}-\omega_{\text{TO,}\psi}^{2}}{\omega_{\text{TO,}\psi}^{2}-i\gamma_{\psi}\omega-\omega^{2}}\right)$ (S6)

where $\psi=\text{ }\parallel,\text{ }\perp$ correspond to the in-plane and out-of-plane phonon modes, respectively. We adopt the established parameter set for bulk hBN [25], with the high-frequency dielectric constants $\varepsilon_{\infty,\text{ }\parallel}=3.03$ and $\varepsilon_{\infty,\text{ }\perp}=4.98$, damping rates $\gamma_{\parallel}=4\text{ cm}^{-1}$ and $\gamma_{\perp}=5\text{ cm}^{-1}$, and key phonon frequencies $\omega_{\text{LO},\text{ }\parallel}=830\text{ cm}^{-1}$, $\omega_{\text{TO},\text{ }\parallel}=780\text{ cm}^{-1}$, $\omega_{\text{TO},\text{ }\perp}=1370\text{ cm}^{-1}$ and $\omega_{\text{LO},\text{ }\perp}=1610\text{ cm}^{-1}$. This form explicitly embeds LO–TO splitting and therefore permits hyperbolic volume polaritons modes when the film is thick enough to sustain internal propagation.

**Figure S8:** Comparison between monolayer and three-dimensional anisotropic hBN models for *N* = 1. (a) Emissivity spectra calculated using the monolayer hBN model and the three-dimensional anisotropic hBN model. (b) Modal fraction analysis of the LP_mono_ branch in the monolayer model. (c) Modal fraction analysis of the MP_bulk_ branch in the quasi-bulk model.

Figure S8 compares the monolayer sheet description with the quasi-bulk description for *N* = 1. As shown in Fig. S8(a), both models capture the same underlying graphene SPP–phonon hybridization, but give different resonance positions and branch ordering because they correspond to different dielectric limits. The monolayer model describes the atomically thin hBN sheet response and is therefore used as the quantitative description for *N* = 1. By contrast, the quasi-bulk model introduces three-dimensional anisotropy, bulk-like LO–TO splitting and finite-thickness polarization channels, which shift the resonances and produce an additional short-wavelength branch. In the monolayer model, LP_mono_ shows a direct exchange between SPP and TO character as *W*_G_ increases, as shown in Fig. S8(b). In the quasi-bulk model, the branch with the closest modal evolution is MP_bulk_, which also evolves from SPP-dominated to TO-dominated character while retaining only a weak LO contribution, as shown in Fig. S8(c). The different *W*_G_ ranges in Figs. S8(b) and (c) reflect a shift of the hybridization region after the LO-related channel is introduced. In the monolayer model, the system is effectively described by two-mode SPP–TO coupling. In the quasi-bulk model, the additional LO channel reorganizes the eigenmode sequence and shifts the effective anti-crossing to a larger *W*_G_. The apparent mismatch between the monolayer and quasi-bulk descriptions therefore arises mainly from branch reordering and anti-crossing displacement, rather than from a change in the underlying plasmon–phonon coupling mechanism.

**Reference**

[1] B. Johns, “Dispersion engineering of infrared epsilon-near-zero modes by strong coupling to optical cavities,” *Nanophotonics*, vol. 12, no. 16, pp. 3301–3312, 2023.
[2] A.-Y. Liu, C.-H. Tseng, K.-I. Lin, and H.-H. Hsiao, “Strong coupling of double resonance designs and epsilon-near-zero modes for mode-matching enhancement of second-harmonic generation,” Nanophotonics, vol. 14, no. 25, pp. 4555–4564, 2025.
[3] P. Xie, W. Wang, and Y. Kivshar, “Resonant light–matter interaction with epsilon-near-zero photonic structures,” *Applied Physics Reviews*, vol. 12, p. 021307, 2025.
[4] D. Wang, Z. Lu, S. Warkander, N. Gupta, Q. Wang, P. Ci, R. Guo, J. Li, A. Javey, J. Yao, F. Wang, and J. Wu, “Long-range optical coupling with epsilon-near-zero materials,” *Nature Communications*, vol. 16, p. 9172, 2025.
[5] S. Choudhary, S. Iqbal, M. Karimi, O. Reshef, M. Z. Alam, and R. W. Boyd, “Strongly Coupled Plasmon Polaritons in Gold and Epsilon-Near-Zero Bifilms,” *ACS Photonics*, vol. 10, pp. 162–169, 2023.
[6] Grigorenko, A. N., Polini, M., and Novoselov, K. S., "Graphene plasmonics," Nat. Photonics, vol. 6, p. 749-758, 2012.
[7] Dai, S., Fei, Z., Ma, Q., et al., "Tunable Phonon Polaritons in Atomically Thin van der Waals Crystals of Boron Nitride," Science, vol. 343, p. 1125-1129, 2014.
[8] Zhang, S., Zhang, H., Xu, T., et al., "Coherent and incoherent damping pathways mediated by strong coupling of two-dimensional atomic crystals with metallic nanogrooves," Phys. Rev. B, vol. 97, p. 235401, 2018.
[9] Chen, Y. K., Wang, B. X., and Zhao, C. Y., "Dual-band spatially-distinguishable metasurface thermal emitter for filterless mid-infrared gas sensing," Int. J. Therm. Sci., vol. 185, p. 108069, 2023.
[10] Qu, Y., Cai, L., Luo, H., et al., "Tunable dual-band thermal emitter consisting of single-sized phase-changing GST nanodisks," Opt. Express, vol. 26, p. 4279, 2018.
[11] Bylinkin, A., Calavalle, F., Barra-Burillo, M., et al., "Dual-Band Coupling of Phonon and Surface Plasmon Polaritons with Vibrational and Electronic Excitations in Molecules," Nano Lett., vol. 23, p. 3985-3993, 2023.
[12] Dao, T. D., Chen, K., Ishii, S., et al., "Infrared Perfect Absorbers Fabricated by Colloidal Mask Etching of Al–Al2O3–Al Trilayers," ACS Photonics, vol. 2, p. 964-970, 2015.
[13] Long, L., Taylor, S., and Wang, L., "Enhanced Infrared Emission by Thermally Switching the Excitation of Magnetic Polariton with Scalable Microstructured VO2 Metasurfaces," ACS Photonics, vol. 7, p. 2219-2227, 2020.
[14] Qu, Y., Li, Q., Cai, L., et al., "Polarization switching of thermal emissions based on plasmonic structures incorporating phase-changing material Ge2Sb2Te5," Opt. Mater. Express, vol. 8, p. 2312, 2018.
[15] Bogaerts, W., De Heyn, P., Van Vaerenbergh, T., et al., "Silicon microring resonators," Laser Photon. Rev., vol. 6, p. 47-73, 2012.
[16] Huang, J. Z., Yin, Z. Q., Wang, S., et al., "Effect of intensity modulator extinction on practical quantum key distribution system," Eur. Phys. J. D, vol. 66, p. 159, 2012.
[17] Makal, A., Benedict, J., Trzop, E., et al., "Restricted Photochemistry in the Molecular Solid State: Structural Changes on Photoexcitation of Cu(I) Phenanthroline Metal-to-Ligand Charge Transfer (MLCT) Complexes by Time-Resolved Diffraction," J. Phys. Chem. A, vol. 116, p. 3359-3365, 2012.
[18] Heßler, A., Wahl, S., Kristensen, P. T., et al., "Nanostructured In3SbTe2 antennas enable switching from sharp dielectric to broad plasmonic resonances," Nanophotonics, vol. 11, p. 3871-3882, 2022.
[19] Yang, S., He, M., Hong, C., et al., "Single-peak and narrow-band mid-infrared thermal emitters driven by mirror-coupled plasmonic quasi-BIC metasurfaces," Optica, vol. 11, p. 305, 2024.
[20] Lyu, X., Heßler, A., Wang, X., et al., "Combining Switchable Phase‐Change Materials and Phase‐Transition Materials for Thermally Regulated Smart Mid‐Infrared Modulators," Adv. Opt. Mater., vol. 9, 2021.
[21] Wang, Z., Clark, J. K., Ho, Y.-L., et al., "Ultranarrow and Wavelength-Tunable Thermal Emission in a Hybrid Metal–Optical Tamm State Structure," ACS Photonics, vol. 7, p. 1569-1576, 2020.
[22] A. Laturia, M. L. Van de Put, and W. G. Vandenberghe, "Dielectric properties of hexagonal boron nitride and transition metal dichalcogenides: from monolayer to bulk," npj 2D Materials and Applications, vol. 2, no. 1, p. 6, 2018.
[23] Kumar, A., Low, T., Fung, K. H., et al., "Tunable Light–Matter Interaction and the Role of Hyperbolicity in Graphene–hBN System," Nano Lett., vol. 15, p. 3172-3180, 2015.
[24] Gil, B., Cassabois, G., Cusco, R., et al., "Boron nitride for excitonics, nano photonics, and quantum technologies," Nanophotonics, vol. 9, p. 3483-3504, 2020.
[25] Zhao, B., Song, J.-H., Brongersma, M., et al., "Atomic-Scale Control of Coherent Thermal Radiation," ACS Photonics, vol. 8, p. 872-878, 2021.
